# Assessment of Gastrointestinal Autonomic Dysfunction: Present and Future Perspectives

**DOI:** 10.3390/jcm10071392

**Published:** 2021-03-31

**Authors:** Ditte S. Kornum, Astrid J. Terkelsen, Davide Bertoli, Mette W. Klinge, Katrine L. Høyer, Huda H. A. Kufaishi, Per Borghammer, Asbjørn M. Drewes, Christina Brock, Klaus Krogh

**Affiliations:** 1 Department of Hepatology and Gastroenterology, Aarhus University Hospital, DK8200 Aarhus, Denmark; meteader@rm.dk (M.W.K.); kathoeye@rm.dk (K.L.H.); klaukrog@rm.dk (K.K.); 2 Steno Diabetes Centre Aarhus, Aarhus University Hospital, DK8200 Aarhus, Denmark; 3 Department of Neurology, Aarhus University Hospital, DK8200 Aarhus, Denmark; astrterk@rm.dk; 4 Mech-Sense, Department of Gastroenterology and Hepatology, Aalborg University Hospital, DK9100 Aalborg, Denmark; d.bertoli@rn.dk (D.B.); amd@rn.dk (A.M.D.); christina.brock@rn.dk (C.B.); 5 Steno Diabetes Centre Copenhagen, Gentofte Hospital, DK2820 Gentofte, Denmark; huda.kufaishi@regionh.dk; 6 Department of Nuclear Medicine and PET-Centre, Aarhus University Hospital, DK8200 Aarhus, Denmark; perborgh@rm.dk; 7 Steno Diabetes Centre North Jutland, Aalborg University Hospital, DK9100 Aalborg, Denmark

**Keywords:** autonomic dysfunction, gastrointestinal, motility, investigations, manometry, breath test, imaging, Parkinson’s disease, diabetes mellitus

## Abstract

The autonomic nervous system delicately regulates the function of several target organs, including the gastrointestinal tract. Thus, nerve lesions or other nerve pathologies may cause autonomic dysfunction (AD). Some of the most common causes of AD are diabetes mellitus and α-synucleinopathies such as Parkinson’s disease. Widespread dysmotility throughout the gastrointestinal tract is a common finding in AD, but no commercially available method exists for direct verification of enteric dysfunction. Thus, assessing segmental enteric physiological function is recommended to aid diagnostics and guide treatment. Several established assessment methods exist, but disadvantages such as lack of standardization, exposure to radiation, advanced data interpretation, or high cost, limit their utility. Emerging methods, including high-resolution colonic manometry, 3D-transit, advanced imaging methods, analysis of gut biopsies, and microbiota, may all assist in the evaluation of gastroenteropathy related to AD. This review provides an overview of established and emerging assessment methods of physiological function within the gut and assessment methods of autonomic neuropathy outside the gut, especially in regards to clinical performance, strengths, and limitations for each method.

## 1. Introduction

Autonomic disorders may involve the parasympathetic, sympathetic, and enteric nervous systems with extensive, multisystemic consequences [1]. Among several other organ manifestations, pan-enteric gastrointestinal (GI) dysmotility is frequently seen [2]. Not only do the motility disturbances contribute to GI symptoms, they may also affect the absorption of medication used to treat the underlying disease [3,4].

Methods for assessment of GI motility are generally applicable across autonomic dysfunction (AD) etiologies despite different underlying pathophysiology. Verification of the extent of GI involvement is important to support diagnosis and guide effective treatment, especially because gastrointestinal symptoms and objective measures correlate poorly [5,6,7,8]. However, commercially available assessment methods have different inherent limitations, and better techniques are needed for evaluating GI dysfunction. Thus, the main focus of this review is to describe established and emerging methods for assessment of enteric dysfunction in patients with AD.

## 2. Clinical Presentation

### 2.1. Autonomic Neuropathy in Neurological Disorders

The autonomic nervous system involves sympathetic and parasympathetic neural structures in the central and peripheral nervous systems that innervate all internal organs [1]. Moreover, and often under-recognized, is the enteric nervous system that is also part of the autonomic nervous system [9]. Centrally, the autonomic nervous system is regulated by areas localized at the forebrain pontomescencephalic and bulbopontine level, and in the spinal cord. The peripheral autonomic nervous system acts via the postganglionic parasympathetic and sympathetic nervous systems, which interact with the enteric nervous system in a complex and delicately coordinated network [10,11]. Thus, central and peripheral nerve lesions and pathology may induce AD [1]. Pure AD can manifest acutely or sub-acutely such as seen in autoimmune autonomic ganglionopathy or treatment-induced neuropathy of diabetes mellitus (DM). The latter can be caused by a too fast downregulation of blood glucose in a dysregulated DM patient [12]. On the other hand, the presentation can be slowly progressing as seen in α-synucleinopathies or neuropathy of various etiologies. α-synucleinopathies are neurodegenerative diseases characterized by abnormal accumulation of aggregates of α-synuclein protein in nerve fibers or glial cells. The main types of α-synucleinopathies are Parkinson’s disease (PD), dementia with Lewy bodies, multiple-system atrophy, and pure autonomic failure [13]. Large and small fiber sensory and autonomic neuropathy is seen in metabolic disorders (DM, hypothyroidism, uremia), cobalamin deficiency, infections, immune-mediated conditions (gammopathies, vasculitis, and coeliac disease), neurotoxic exposure (alcoholism, and pharmacological treatment), and in hereditary conditions (hereditary sensory and autonomic neuropathy, Fabry’s disease, and hereditary transthyretin-mediated amyloidosis) [14]. Autonomic dysfunction is also seen in patients with postural orthostatic tachycardia syndrome (POTS) defined by an abnormal increase in heart rate of at least 30 beats/min within 10 min of standing or during a tilt table test. The rise in heart rate is seen in the absence of orthostatic hypotension and symptoms of orthostatic intolerance must be present for at least 6 months [15]. POTS has been associated with small fiber neuropathy, Ehlers–Danlos syndrome and mast cell activation syndrome [16,17].

### 2.2. Clinical Presentation of Autonomic Neuropathy in General

The symptoms of autonomic neuropathy are numerous and the condition is multisystemic due to the extensive parasympathetic and sympathetic innervation of multiple organs and structures such as the cardiovascular, gastrointestinal, thermoregulatory, respiratory, urogenital, pupillomotor, and sudomotor systems [2]. Thus, diagnosis, treatment, and follow-up may involve multiple specialties. Parasympathetic dysfunction may cause the sicca syndrome with dry eyes and mouth, light intolerance due to dilated non-responding pupils, urine retention, erectile dysfunction, resting tachycardia, and reduced GI motility. Sympathetic dysfunction is characterized by miotic pupils, orthostatic intolerance with dizziness or syncope, exercise intolerance, anhidrosis, and heat intolerance [18]. GI dysfunction may cause gastroparesis and enteropathy with constipation, diarrhea, and fecal incontinence, and may affect absorption of oral medication, see below.

Recognizing AD is important because of the increased morbidity and mortality associated with reduced heart rate variability, arrhythmias, increased blood pressure variability, and neurogenic orthostatic hypotension [19,20]. Acute development of AD can be the first sign of an underlying paraneoplastic condition. Furthermore, early recognition is important to ensure early initiation of conservative or pharmacological treatments targeting orthostatic or postprandial hypotension, supine hypertension, erectile dysfunction, and gastroenteropathy as these conditions may have a negative impact on the quality of life if left untreated. Finally, autonomic testing can monitor the course of dysautonomia and the response to treatment.

### 2.3. Clinical Presentation of Gastrointestinal Autonomic Neuropathy

Studies of GI function in patients with AD have mainly included patients with DM or PD. However, pan-enteric autonomic neuropathy is also seen in the less commonly described etiologies, and principles for clinical evaluation and treatment will be largely similar across etiologies. All segments of the GI tract may be affected, contributing to a highly variable inter-individual clinical presentation and intra-individual symptom fluctuation with time, the latter especially seen in patients with DM [21]. Common GI symptoms, such as dysphagia, nausea, vomiting, bloating, early satiety, abdominal pain, constipation, diarrhea, weight loss, and fecal incontinence may be present, combined or solitary, and they may substantially affect the quality of life [22,23,24]. In patients with DM, symptoms of gastroparesis are present in up to 18%, diarrhea in 20%, and constipation in up to 60%. Furthermore, fecal incontinence is frequently reported [25,26]. The prevalence of symptoms of gastroparesis and constipation in PD reaches 50%. Furthermore, 72% have anorectal dysfunction expressed as straining for defecation, but also incomplete emptying, with symptoms becoming more severe during disease progression [27,28]. Constipation is reported in 50% of patients with pure autonomic failure and in up to 82% of patients with multiple system atrophy [3]. Orthostatic symptoms in POTS often coexist with severe GI symptoms, with nausea, abdominal pain and constipation reported in more than 70% [29,30]. Prominent multi-segmental GI symptoms are also commonly seen in the hypermobile Ehlers–Danlos syndrome and in the mast cell activation syndrome, which is relevant as a differential diagnosis [16,17]. However, prevalence measures vary across studies in all the above-mentioned disorders. While several AD etiologies are associated with GI symptoms, studies on motility across multiple GI segments are primarily performed in PD and DM. Thus, motility disturbances in PD and DM will gain most attention in this review, but dysmotility findings in other diseases will be mentioned when available.

**Figure 1:**
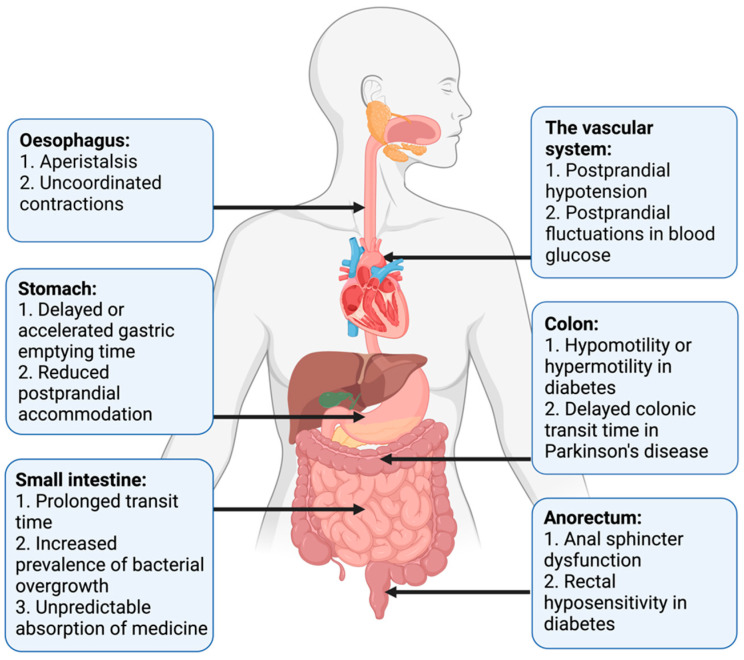
Motility disturbances related to autonomic dysfunction in each gastrointestinal segment.

Pan-enteric dysmotility has been documented, and the abnormalities in each segment of the GI tract are presented in Figure 1. Aperistalsis and uncoordinated contractions are common in the esophagus [7,31]. Gastric dysmotility presents as delayed or accelerated gastric emptying time and reduced postprandial accommodation [21,30]. Dysmotility, prolonged transit time, and a higher prevalence of small intestinal bacterial overgrowth (SIBO) are seen within the small intestine [32,33]. Delayed colonic transit time is frequently seen in PD and primarily caused by a combination of slow transit constipation and anorectal outlet obstruction [27]. Anorectal dysfunction in PD is primarily due to dystonia and pathological contractions of the external sphincter during defecation [27]. Both colonic hypo- and hypermotility have been shown in DM and a dysfunctional internal sphincter combined with rectal hyposensitivity contributes to fecal incontinence [34,35,36].

Widespread dysmotility and varying transit times, especially prolonged gastric emptying time, can make the absorption of oral medication unpredictable and reduce the effectiveness of some drugs [3,4]. Additionally, abnormal postprandial fluctuations in blood glucose, related to a mismatch between insulin administration and food availability in the small intestine, may be harmful to patients with DM [21]. Postprandial hypotension, mainly related to autonomic neuropathy, is also more frequent in patients with DM than in healthy controls [37].

No commercially available in vivo diagnostic test of enteric neuropathy exists. Furthermore, GI symptoms are generally not predictive of the objective motility dysfunction, with objective dysmotility occurring more frequently than subjective symptoms. This necessitates objective assessment to verify the extent of GI dysmotility to support the diagnosis of enteric neuropathy and guide treatment [5,6,7,8]. However, even though a verification of GI dysmotility in a patient with AD significantly increases the likelihood of enteric neuropathy, some patients may have enteric neuropathy despite normal motility measurements.

The underlying pathophysiology of AD varies across patient groups, but assessment methods of the pan-enteric dysfunction are overall identical. Thus, established and emerging methods for assessment of gut function in autonomic disorders and the most relevant general assessment methods of autonomic neuropathy will be reviewed below. The assessment-guided treatment approach will be described at the end of this review.

## 3. Established Methods for Assessment of Gastroenteropathy

### 3.1. Exclusion of Differential Diagnoses

When enteric neuropathy is suspected in a patient with an autonomic disorder, the primary approach is to exclude other plausible causes of the gastrointestinal symptoms, such as gastrointestinal cancer, inflammatory bowel disease, exocrine pancreas insufficiency, bile acid malabsorption, coeliac disease, and porphyria. Furthermore, it is important to substitute medication if side effects are suspected to be the cause of GI symptoms.

### 3.2. Assessment of Symptoms

In spite of several scoring systems being used in the literature, no questionnaire has been validated specifically for assessment of AD-related gastroenteropathy, except for the GI sub-score within the Composite Autonomic Symptom Score (COMPASS-31) questionnaire, see Section 5 [38].

The Gastroparesis Cardinal Symptom Index is a sub-score in the larger questionnaire PAGI-SYM (patient assessment of upper gastrointestinal disorders-symptom severity index) [39]. It is a symptom severity scale assessing gastroparesis and consists of nine items grouped into three subscales including nausea/vomiting, postprandial fullness/early satiety, and bloating. The severity of each symptom is rated on a Likert scale ranging from 0 (no symptoms) to 5 (very severe symptoms), and the recall period is two weeks. The Gastroparesis Cardinal Symptom Index is reliable, valid, and responsive to change [40,41]. However, gastroparesis can be asymptomatic and previous studies suggest that delayed gastric emptying cannot be predicted by the severity of symptoms alone [42,43].

The Gastrointestinal Symptom Rating Scale is a well-validated, responsive, and reliable instrument for assessing GI symptoms. It has been used in several clinical trials mainly for dyspepsia and gastroesophageal reflux disease, but also in patients with DM [44,45,46]. It consists of 15 items covering five symptom clusters: reflux, abdominal pain, indigestion, diarrhea, and constipation. A 7-point Likert-type response scale is used to grade the severity of symptoms, ranging from 1 (no symptoms) to 7 (very troublesome symptoms), and the recall period is the past week [44].

Specific constipation scoring systems have also been used in autonomic disorders. The Cleveland Constipation Score consists of eight items, and a total score above 15 represents constipation. Symptoms are graduated from mild to severe, which allows for monitoring of symptom fluctuation [47]. The ROME IV criteria for constipation are commonly used to define functional constipation and combine a detailed description of colonic and anorectal symptoms [48]. They are, however, not directly applicable in patients with AD-related gastroenteropathy.

The Diabetes Bowel Symptom Questionnaire is validated for assessment of GI symptoms, glycemic control, and quality of life in patients with DM, but has been used only sporadically [49]. Questionnaires addressing the broad spectrum of non-motor-symptoms in PD have been developed. These do not cover pan-enteric GI dysfunction in detail but are useful as screening tools [27].

### 3.3. Tests of Esophageal Motility

Within recent years, *high-resolution esophageal manometry* has been the method of choice for examining esophageal dysfunction in neurological disorders [50]. When an upper endoscopy with biopsies does not explain the underlying cause of symptoms such as dysphagia and regurgitation, esophageal manometry may be performed. The manometry catheter contains up to 36 pressure sensors distributed 1 cm apart. These sensors provide spatiotemporal, topographic maps of the propagating motor patterns by measuring amplitudes of contractile events within the regions of interest [51]. The clinical performance and interpretation of these data can be challenging. Therefore, when high-resolution esophageal manometry is used to assess AD, it is normally restricted to specialized centers [52]. Esophageal motor dysfunction is present in half of all patients with type 1 DM and dysphagia [53]. In addition, esophageal dysmotility is frequently seen in the α-synucleinopathies, most often as generally reduced peristalsis with ineffective swallows [31,33]. Absent or impaired esophageal activity is documented in POTS with conventional esophageal manometry and with high-resolution esophageal manometry in the Ehlers–Danlos Syndrome, hypermobility type [54,55].

The *modified barium swallowing test* can also be utilized in the diagnosis of these disorders. This examination permits the dynamic visualization of content movements through the upper GI system in real time with the use of videofluoroscopy [56]. The role of the modified barium swallowing test is not limited to the diagnose of dysmotility but can add to the understanding of the physiologic swallowing deficit, which can be useful to maximize the benefit of swallowing therapy [57]. Unfortunately, this examination suffers a highly variable inter- and intra-rater reliability, requires considerable resources, and is associated to radiation exposure as well as aspiration risks [56,58]. The modified barium swallowing test demonstrated slower initiation of airway closure in patients with PD [57]. The test is utilized in the diagnostics of esophageal dysmotility in other causes of autonomic dysfunction as well but the literature on this area is still scarce [59].

### 3.4. Gastric Emptying Tests

Assessment of gastric emptying time is indicated when patients with an autonomic disorder suffer from nausea, early satiety, lack of appetite, vomiting, postprandial pain, unpredictable absorption of orally administered medication, or large postprandial blood glucose fluctuations in DM. Various assessment techniques exist, and the choice of method primarily depends on its availability at each center performing the procedure.

*Gastric emptying scintigraphy* is the gold standard for measuring gastric emptying time. An ingested, standardized radiolabeled meal is followed by sequential gamma camera images at minimum 0, 1, 2, and 4 h after meal ingestion [60,61]. The region of interest is drawn manually on each image, and the percentage of activity remaining in the stomach at each time-point expresses gastric emptying [62]. The advantage of this technique is its effective and non-invasive character that does not interfere with normal gastric motility. However, exposure to radiation, high cost, and limited availability are major drawbacks for all scintigraphic measurements. Scintigraphy has shown delayed or rapid gastric emptying time in patients with DM, and delayed gastric emptying time in patients with multiple system atrophy and PD [8,63]. In patients with POTS gastric emptying time is more frequently rapid than delayed [64].

*Gastric emptying breath test* is a simple, inexpensive, non-invasive, and radiation-free technique to measure gastric emptying time. A solid meal containing the non-radioactive isotope ^13^C is ingested and rapidly absorbed when it enters the small intestine. Gastric emptying is the rate-limiting step in the metabolic pathway for ^13^CO_2_; and after metabolization in the liver, ^13^CO_2_ is exhaled through the respiratory tract, whereby the accumulation of ^13^CO_2_ in the breath samples indirectly reflects gastric emptying time [65]. Gastric emptying time measures from the gastric emptying breath test are reproducible and correlate with findings from gastric emptying scintigraphy in patients with DM [21,66]. The disadvantage of this technique is the multiple steps required from ingestion to exhalation, which may make the test less accurate. Normal lung and liver function are also a prerequisite. Patients with multiple system atrophy have significantly prolonged gastric emptying time when investigated with gastric emptying breath test [67]. Unfortunately, a recent meta-analysis showed that gastric emptying time obtained with gastric emptying scintigraphy and gastric emptying breath test correlate poorly in patients with PD, and the validity of the test is questioned in this disease [68].

### 3.5. Assessment of Gastric and Small Intestinal Motility

*Antropyloroduodenal manometry* can distinguish abnormal from normal motility patterns within the distal stomach, pylorus, and duodenum. The method is performed only at a few and highly specialized centers and usually as a supplement to gastric emptying tests. Specific motility patterns can be demonstrated in both fasting and postprandial states. However, different disorders may share common dysmotility patterns. Antropyloroduodenal manometry is in general seen as a valuable diagnostic tool and can guide treatment in various motility disorders [69]. The method has been used in patients with DM, but the clinical evidence is otherwise sparse in gastroenteropathy related to AD [69,70].

Usually, water-perfused or solid-state catheters are used with pressure sensors spaced 5–10 cm in the duodenal region and 0.5–1 cm in the antral and pyloric region. The recording period is often 6 h and includes the ingestion of a meal. However, ambulatory recording can be performed over 24 h, which may reduce variability among individuals but increases the risk of catheter displacement [71]. The method is reproducible and the interobserver agreement is comparable to that of other commonly used methods [69,72]. Normative values are available [73]. However, it may be unpleasant for the patients to carry the catheter, and expertise is needed to perform the investigations and to analyze data. Application of the high-resolution esophageal manometry catheter in the antropyloroduodenal region can demonstrate more detailed motility patterns than antropyloroduodenal manometry, but these catheters are expensive and more sensitive to external noise, such as cough and movements [74].

### 3.6. Tests of Small Intestinal and Colonic Transit

Assessment of small intestinal or colonic transit times is mainly indicated in patients with abdominal bloating and pain or in patients with symptoms of constipation. It may also be relevant in patients where symptoms of constipation or diarrhea coexist in order to obtain information on the underlying physiology and aid the choice of treatment, see Section 6.

*Scintigraphy* is established for measuring transit times through the small bowel, colon, and whole gut [75]. The basic principles are similar to those of gastric emptying scintigraphy. However, for small bowel transit time gamma images are continued for 6 h after ingestion, and single images at 24, 48, and 72 h are used to determine colonic transit time [62]. Only a few normative data with a wide normal range are available for small bowel transit time and the interpretation is potentially affected by abnormal gastric or colonic motility. Lack of standardization in clinical practice and time-consuming protocols are drawbacks for intestinal scintigraphy in general [61]. Thus, the method has only gained limited use in AD-related gastroenteropathies [76].

*Radio-opaque markers* are the most commonly used method for assessment of whole gut transit time, which in clinical practice can be seen as an approximation of colonic transit time. The method is simple, repeatable, well-tolerated, inexpensive, and easy to perform. In addition, good correlation has been demonstrated for colonic transit time measured with radio-opaque markers, Wireless Motility Capsule, and scintigraphy [77,78]. Usually, the markers are taken on a single day and visualized by an X-ray on day 5. If quantitative data are needed, a capsule containing 10 markers is ingested on 6 consecutive days with an abdominal X-ray on day 7 [79,80]. Estimation of segmental colonic transit times also requires ingestion of radio-opaque markers at consecutive days, and patient compliance has to be optimal. Other limitations are the radiation exposure and the lack of method standardization between centers, which challenges comparison of the results [61]. Assessment with radio-opaque markers in patients with PD, multiple system atrophy and DM showed significantly prolonged colonic transit time, especially within the left and rectosigmoid colon [5,27,81,82].

*Hydrogen and methane breath tests* can quantify orocecal transit time as a combined measure of gastric and small intestinal transit. The test is usually used as a supplement to assessment of colonic transit time with radiopaque markers and mainly in patients with bloating, abdominal discomfort, or diarrhea. When in contact with colonic bacteria, ingested non-absorbable carbohydrates undergo fermentation and release gases, such as hydrogen and methane, which are excreted through respiration within 3 min. Orocecal transit time is defined as the time interval between oral intake of carbohydrates (often 10 g lactulose) and a registered peak in expired gases by gas chromatography. The hydrogen and methane breath tests are simple, non-invasive, inexpensive, and without exposure to radiation. However, the correlation between the hydrogen breath test and scintigraphy is variable [83,84]. In addition, several other sources of error exist. The natural osmotic activity of lactulose potentially accelerates small intestinal transit and decelerates gastric emptying. The presence of SIBO may complicate the interpretation of orocecal transit time [61]. In both DM and PD, orocecal transit time was significantly prolonged compared with healthy controls when using the hydrogen breath test [85,86].

### 3.7. Assessment of Small Intestinal Bacterial Overgrowth

Patients with intestinal dysmotility, and among these patients with AD-related gastroenteropathy, are predisposed to SIBO [24,87]. The prevalence of SIBO depends on the choice of diagnostic method [32,88]. Assessment of this condition is primarily needed when abdominal discomfort, bloating, and diarrhea are present in patients with AD. The most valid method for diagnosing SIBO is a luminal, jejunal aspirate for culture retrieved by endoscopy, but this method is invasive, subject to contamination, and may underestimate the intraluminal amount of microbiota. In addition to their use for assessment of orocecal transit time, *hydrogen and methane breath tests* are frequently used as an indirect and non-invasive method to detect SIBO. When SIBO is present, an early peak of expired hydrogen or methane gas is recognized due to fermentation within the small intestine [32]. A North American consensus provides a practical guide to a standardized performance and interpretation of breath tests, and these tests are widely used in clinical practice [89]. However, recent studies have questioned the utility of breath tests for diagnosing SIBO [90]. Simultaneously performed scintigraphy and breath test showed that rapid orocecal transit time and hereby early colonic fermentation with production of hydrogen or methane gas could erroneously be interpreted as SIBO [91]. Jejunal aspirates for culture did not correlate well with the breath test, and in general methods for diagnosing SIBO lack sensitivity, specificity, reproducibility, and standardization [90].

### 3.8. Tests of Anorectal Motility

*High-resolution anorectal manometry* and *high-definition anorectal manometry* are increasingly used in clinical practice to evaluate continence and regulation of defecation, primarily in patients with either difficult evacuation of stools or fecal incontinence who do not respond to standard treatment modalities [92]. A consensus guideline for standardization of the methods was recently published [93]. Compared with conventional manometry, additional pressure sensors are closely incorporated within either a solid-state or a water-perfused catheter (often ≥8 sensors). A high-definition rigid catheter containing 256 pressure sensors arranged in a circumferential grid has also been developed [92,94]. In combination with anorectal sensibility tests or other diagnostic investigations, contractions in the distal rectum and anal canal in response to various stimuli may establish a diagnosis and direct different treatment modalities [93]. Normative values based on large datasets exist for both high-resolution and high-definition anorectal manometry [95,96]. Limitations to both techniques are their fragility and costs. Moreover, data analysis is challenging, limiting their use to investigation at specialized centers. High-resolution anorectal manometry has been used to evaluate anorectal dysfunction in PD, especially revealing dystonic contractions in the external anal sphincter as a pathophysiological mechanism for unsuccessful attempts of defecation [97,98]. Reduced anorectal sensibility and internal sphincter dysfunction contribute to fecal incontinence in patients with DM [35].

### 3.9. Whole Gut Assessment

**Figure 2:**
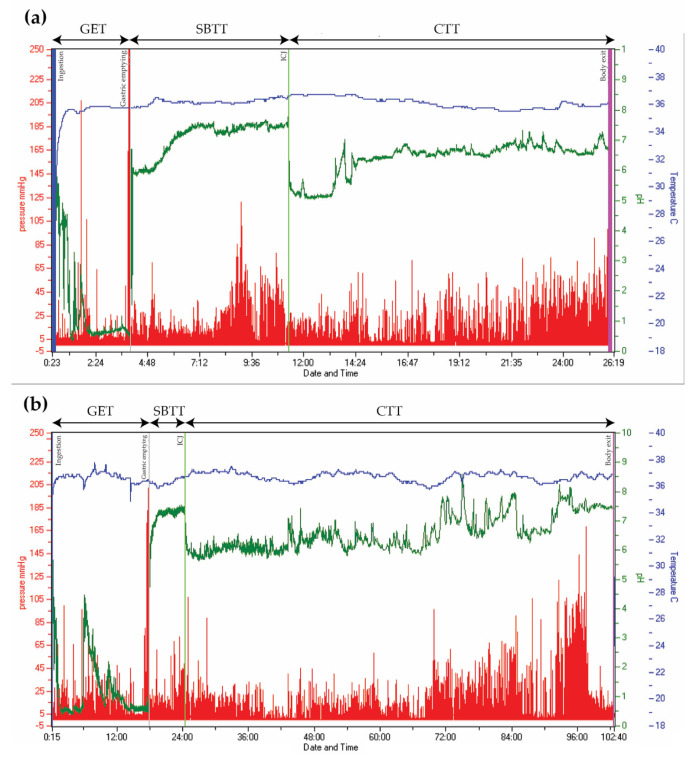
Wireless Motility Capsule recordings from two patients with type 2 diabetes. Time is displayed on the x-axis, pressure on the left y-axis (red), pH on the right y-axis (green), and temperature on the right y-axis (blue). (**a**) Normal transit times. (**b**) Delayed gastric emptying time (18 h) and colonic transit time (78 h). (GET = Gastric emptying time. SBTT = Small bowel transit time. CTT = Colonic transit time. ICJ = Ileocolic junction).

When pan-enteric dysmotility is suspected, often due to combined upper and lower GI symptoms, *the Wireless Motility Capsule* (Smartpill Monitoring System; Medtronic) is considered the method of choice. An ingested capsule measures pH, intraluminal pressure, and temperature while it passes through the GI tract and transmits this information to a wireless receiver [99]. Accurate measures of the total and regional transit times are provided by using specific pH changes as a surrogate for GI physiological landmarks and temperature to verify expulsion, as seen in Figure 2 [36,99]. The advantages of this test are the availability of substantive normative data and its ambulatory, non-invasive, and radiation-free character [100,101]. Results from the wireless motility capsule correlate with established methods for measuring regional and whole gut transit times [102,103,104]. Lack of information on segmental colonic transit times is a drawback for the wireless motility capsule investigation. In addition, it only provides information on localized intestinal pressure changes rather than detecting a peristaltic wave, whereas external noise, such as a cough and body movements, can be misinterpreted as bowel movements. The SmartBar, ingested along with the wireless motility capsule, has a high sugar content, which may induce hyperglycemia and by this a slower gastric emptying in patients with DM [105]. Evidence suggests multi-segmental dysmotility in the GI system of both patients with POTS and DM, and a recent study showed that test results led to treatment changes in 73% of patients with DM [6,106]. In patients with PD, multi-segmental delayed transit times determined by the wireless motility capsule can also guide treatment [107]. Hence, evaluation of the entire GI tract with only one examination seems like a reasonable choice in AD-related gastroenteropathy [6,36,108].

Pan-enteric assessment methods, such as the wireless motility capsule, are not widely available. Thus, the initial assessment of motility-disturbances is commonly performed by combining a gastric emptying test (for example the gastric emptying scintigraphy), a breath test for SIBO (for example the hydrogen and methane breath tests) and a test of colonic transit time (for example the radio-opaque markers). Furthermore, guided by symptoms and objective motility findings, it may be relevant to perform one of the mentioned manometric investigations. The influence of the assessment methods on management will be reviewed briefly in Section 6.

## 4. Emerging Methods for Assessment of Gastroenteropathy

### 4.1. Whole Gut Assessment

*The electromagnetic 3D-Transit* system (3D-Transit, Motilis Medica SA, Lausanne, Switzerland) is an ambulatory, minimally invasive, and capsule-based technique, which presents similarities to the wireless motility capsule by providing information on regional and whole gut transit times. As the only available technique, the 3D-Transit system can also be used to assess segmental colonic transit times and simultaneously provide a detailed assessment of contraction patterns in a precise anatomical location [109]. A detector plate worn in a belt around the abdomen detects the electromagnetic field emitted by an ingested electromagnetic capsule. The electromagnetic field is converted into space-time coordinates, with three spatial coordinates (x, y, and z) representing the three-dimensional capsule position within the GI system, and two orientational coordinates (ϕ, θ) representing the angular rotation of the capsule in two directions. An accelerometer within the detector plate and a thoracic belt detect postural changes and breathing artefacts to be filtered out in the data analysis [109,110]. Propagation of luminal content within the GI tract is expressed by the change in orientation of the capsules and capsule movement velocity. The characteristic contraction frequency in each GI segment is determined by angular rotations of the capsule providing information about anatomical landmarks [111,112]. The 3D-Transit system can track three capsules simultaneously without interference, and the measures of transit times are found to be valid, reproducible, and comparable to transit times measured with radio-opaque markers [110]. Normative data on healthy subjects are available and comparable to normative data on transit times from the wireless motility capsule [100,113,114]. The main drawbacks of using this pan-enteric, diagnostic tool is the time-consuming and challenging data analysis, no CE-marking, and no availability outside research settings [112].

3D-Transit has been applied to assess transit times and contraction patterns in various GI disorders and among these in patients with gastroenteropathy related to AD [112]. Patients with type 1 DM are shown to have prolonged gastric emptying time, colonic transit time, and whole gut transit time mainly due to an increased number of retrograde movements within the right colon [115]. Furthermore, widespread prolonged transit times, especially through the small intestine and right colon, and fewer antegrade mass movements have been found in PD [116].

### 4.2. Tests of Colorectal Contractions

*High-resolution colonic manometry* provides the most precise and detailed description of motor patterns within the colon and has essentially contributed to the understanding of normal colonic physiology [117]. The catheters used are either water-perfused, solid-state, or fiber-optic, with sensors spaced 1–3 cm apart to increase the resolution. The contractile activity is presented by spatiotemporal, color-graded, typographical maps, which allows detection of pressure amplitudes and movements in both antegrade and retrograde directions [118]. On the other hand, high-resolution colonic manometry is time-consuming and lacks standardization in respect to the type of catheter, number of sensors, distance between sensors, composition of ingested meals, use of anesthetics, and length of measurements. The technique involves colonoscopy for placement of the catheter and therefore a need for bowel preparation, which can affect colonic motor activity [118]. Data analysis requires an experienced investigator. However, a recently published consensus statement labelling colonic motor activity provides a common ground for future data analysis [119]. High-resolution colonic manometry is still primarily used for research but is a promising clinical tool for assessment of colonic motor activity, also in patients with gastroenteropathy and AD.

### 4.3. Imaging

#### 4.3.1. Computed Tomography (CT)

In clinical practice, X-ray is the standard test to identify severe fecal retention, but an objective volume estimation technique to be used as a surrogate for the colonic function is lacking. Due to the increased prevalence of constipation in autonomic disorders, combined with alterations in the intestinal tissue, organ sizes may change [5,120,121].

**Figure 3:**
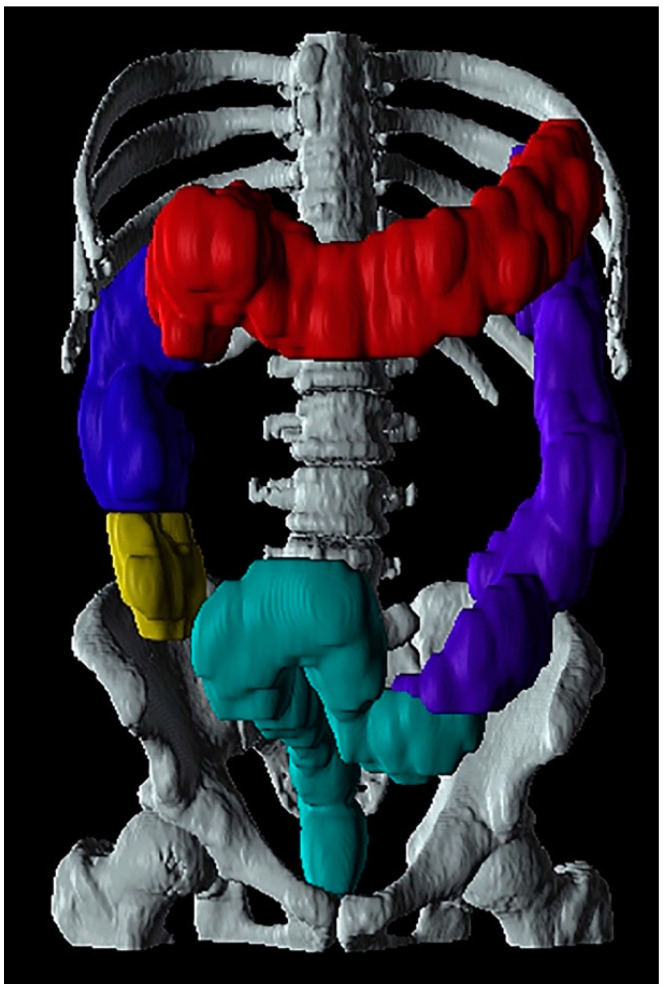
Example of colonic volumes from a computed tomography scan. Yellow: cecum, blue: ascending colon, red: transversal colon, purple: descending colon and turquoise: rectosigmoid colon. Used with permission from M. Klinge, Dissertation, January 2020.

A recent study defined the colonic and small intestinal volumes from CT scans in patients with type 1 DM, finding an increased volume [122]. Additionally, an increased intestinal volume was seen in the transverse colonic and rectosigmoid segments of patients with PD, representing the combination of slow transit constipation and outlet obstruction [5]. CT scans are widely available in all hospitals and often performed in clinical practice. However, ionizing radiation used in CT scans limits their use, especially in pediatric patients and pregnant women [123]. The data analysis in CT-extracted intestinal volumes is time-consuming and currently not applicable in a clinical setting. Colonic volumes from a CT scan are presented in Figure 3.

#### 4.3.2. Ultrasound Imaging

*Ultrasound imaging* is a useful radiation-free option for showing various diameters of the gut. However, the limitations of ultrasound imaging include difficulty in examining the deep abdominal loops, and a skilled radiologist is needed to obtain a sufficient result [123]. Ultrasound imaging is only sparsely used in gastroenteropathy related to AD [124].

#### 4.3.3. Magnetic Resonance Imaging (MRI)

**Figure 4:**
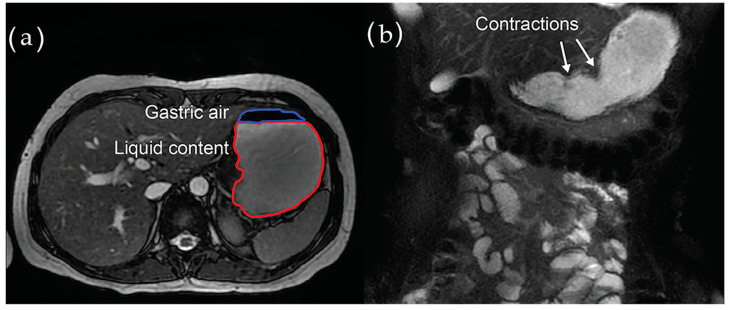
Magnetic resonance imaging of the stomach. (**a**) Gastric air volume and liquid content volume obtained in the segmentation process. (**b**) Contraction waves observed and quantified in the coronal plane.

Visualization of the GI tract with *MRI* has advanced significantly during the past decade. MRI techniques provide morpho-functional information while being feasible and non-invasive [125,126]. MRI has been applied in patients with DM or PD, primarily for assessment of gastric motility [127,128,129,130]. At present, MRI holds promise for assessment of gastric function in terms of accommodation, motility indexes, gastric emptying velocity, and volumetric strain, while simultaneously describing the anatomy of the organ [127,131,132]. Gastric contraction waves and measurement of gastric volume obtained with MRI are seen in Figure 4. The small intestine is an especially challenging organ for imaging methods. However, MRI allows imaging of the small bowel wall, small intestinal lumen, and the surroundings in one scan without ionizing radiation. Enteral contrast agents can be added for better delineation of the intestinal wall [123]. Furthermore, colonic and rectosigmoid volumes can be assessed.

While promising, MRI examinations of GI volumes are not yet used in clinical practice because they are relatively expensive and require highly trained examiners.

#### 4.3.4. ^11^C-Donepezil Positron Emission Tomography/Computed Tomography (PET/CT)

**Figure 5:**
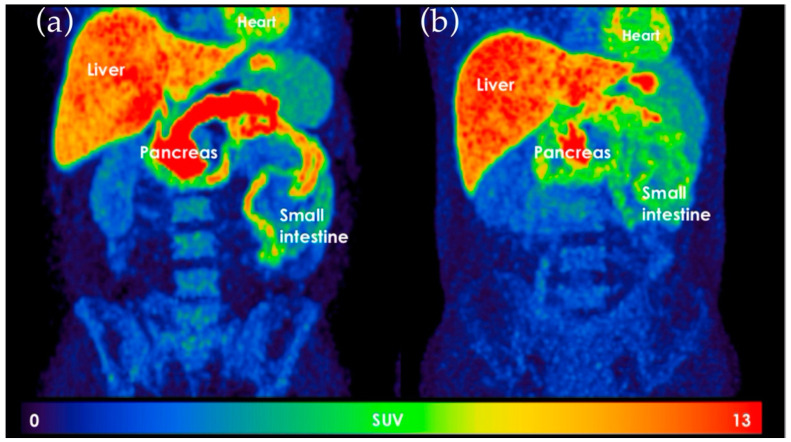
^11^C-donepezil positron emission tomography images. (**a**) Healthy control. (**b**) Patient with diabetes mellitus. Notice the difference in the standard value uptake in the pancreas and the small intestine. The picture is used with permission from Klinge, et al., 2020.

^11^C-donepezil PET/CT scan visualizes the cholinergic innervation of the GI tract in vivo and may potentially fill the need for a future method to assess the severity of GI autonomic neuropathy [33]. The radioactive tracer (^11^C-donepezil) is injected, and standardized uptake values in the internal organs are recorded. The scan is performed without bowel preparation in near-normal conditions. The validity of this imaging method to detect intestinal parasympathetic denervation is confirmed by a significantly decreased ^11^C-donepezil intestinal signal in patients after truncal vagotomy [133]. Patients with early PD have a significant signal loss of ^11^C-donepezil within the intestine as the result of cholinergic denervation [134]. This intestinal denervation corresponds well with the degree of α-synuclein in parasympathetic neurons in PD [33,135]. Parasympathetic intestinal denervation indicated by reduced ^11^C-donepezil uptake is also found in patients with DM [122]. This is presented in Figure 5. The disadvantages of the method are that it is only performed in very few centers and requires comprehensive data analysis.

### 4.4. Gut Biopsies

Another way to diagnose GI autonomic neuropathy is to analyze intestinal biopsies. The optimal way to quantify enteric neurons is by obtaining whole-mount preparations to visualize both the submucosal plexus and the myenteric plexus by immunohistochemical neuronal markers [136]. Furthermore, it is possible to analyze the density of enteric neurons in formalin-fixed, paraffin-embedded biopsies, but the validity of this method remains debated. This is partly due to a lack of normative data and standardized quantitative methods for counting the neurons and the absence of a clear-cut definition of a ganglion [137]. Another limitation is that neurons are almost entirely drawn from the submucosa, unless full-wall biopsies are taken. Neurons of the enteric nervous system can partly be visualized by light microscope when stained with neuronal markers like neuron-specific enolase and synaptophysin. In addition, mast cells and ICC in enteric biopsies can be visualized and counted microscopically by staining with C-kit/CD117 [16,138] A new approach is non-invasive mapping of full-thickness segments of the gut and identification and quantification of ganglia of the enteric nervous system by a technique named *optical coherence microscopy* [139].

In a recent study, jejunal full-thickness biopsies were collected from patients suffering from severe gut dysmotility, either by laparoscopy or by conventional abdominal laparotomy. By quantifying the inter-ganglionic distance between neighboring myenteric ganglia and the number of neurons per ganglion in the myenteric and submucosal plexus, the authors showed that patients with enteric dysmotility had significantly fewer myenteric and submucosal neurons [140]. The methodology has been refined, and a new technique has utilized the evaluation of standard submucosal biopsies. The submucosa is micro-dissected and fixed for later immunofluorescence staining to characterize the morphometry of the plexus and the enteric glial cell. Immunohistochemically, the neurons of the enteric nervous system are visualized by a light microscope using standard protocols of staining [138]. Similarly, mucosal biopsies from the stomach of patients with DM have been used for quantifying gastric mucosal nerve fiber length and volume density [141]. Finally, α-synucleinopathies immunostaining of colonic submucosal biopsies has shown aggregation of α-synuclein in the enteric nervous system and holds promise as an early diagnostic marker for PD [24,142].

**Figure 6:**
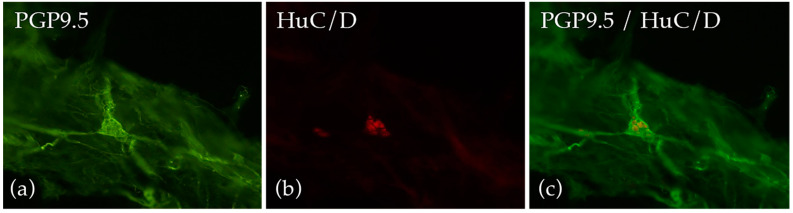
Morphological analysis on human submucosal plexi from colonic standard submucosal biopsies. The used primary antibodies encounter two general pan-neuronal markers, i.e., (**a**) PGP9.5 recognizing perikarya and nerve fibers and (**b**) HuC/D detecting only neuronal cell bodies for quantitative analysis. (**c**) The two neuronal markers, PGP9.5 and HuC/D, used simultaneously. Giancola, Brock and de Giorgio, unpublished data.

Taken together, several newly established techniques have been developed in which the submucosa and related plexuses are isolated from the mucosa in endoscopically obtained surface biopsies and can be used to evaluate the enteric nervous system in health and disease [143,144]. At present, the methods are almost entirely for research purposes. A morphological analysis of a submucosal biopsy is presented in Figure 6.

### 4.5. Assessment of the Human Gut Microbiota

The human gut microbiota consists of trillions of symbiotic bacterial, viral, and fungal microorganisms [145]. New techniques for assessment of the human gut microbiome have facilitated large-scale analysis of the microbial community. Genetic analysis is based on sequence divergences of small subunit ribosomal RNA (16S rRNA). It can provide information on microbial diversity, qualitative and quantitative information on bacterial species, and changes in gut microbiota related to disease [146].

Studies have demonstrated that gut microbiota participate in many aspects of human physiology including the development of the immune system, energy metabolism, and activity of the nervous system [147]. Neurological diseases such as PD present a different gut microbiota composition than encountered in healthy controls [148,149]. Studies show an association between PD and the abundance of certain microbiota. However, it is not yet known whether it is the microbiota or the microbiota-derived metabolites that has an impact on the disease [150]. Microbial metabolites such as short chain fatty acids, which are considered neuro-reactive, are produced by the microbiota, and may enter the systemic circulation. Studies have shown that PD patients have lower levels of fecal short chain fatty acids, which may have a protective effect against the development of PD [151]. Further studies are required to determine the role between the presence or absence of specific microbiota and microbiota-derived metabolites.

Patients with type 1 DM have a less diverse and less stable gut microbiome than healthy controls [152,153]. Findings have not been conclusive, but most studies have found reduced diversity of the intestinal bacterial community and an increased proportion of Bacteroides [154]. Studies have also focused on the intestinal epithelial barrier which prevents food antigens and bacteria from leaving the gut lumen and entering the body leading to a systemic immune response. Disruption and increased permeability of the intestinal barrier have been shown in intestinal autoimmune diseases as well as type 1 DM [155,156]. Preclinical studies support the hypothesis that specific features of the microbiota give rise to impaired intestinal permeability [157], which further influences T cell autoimmunity and B-lymphocytes. This may lead to beta-cell destruction and type 1 DM. However, it has not been confirmed whether alterations in gut microbiota and increased gut permeability are causally related to the pathophysiology or merely a consequence of disease.

Microbiome analysis on the human gut microbiota has significantly improved our knowledge of gut microbiota composition and diversity. An understanding of the human gut microbial diversity in different types of disorders might provide insight into the clinical application in diagnosis and treatments of disease. However, a significant association between microbial patterns and disease initiation or progression has yet to be unveiled.

The above mentioned established and emerging methods for assessment of gut function are summarized in Table 1.

## 5. Assessment of Autonomic Neuropathy Outside the Gut

Since no commercially available in-vivo diagnostic test of enteric neuropathy exists and the described tests of GI physiological function all have significant limitations, some patients with symptoms of autonomic GI dysfunction may benefit from assessment of extraintestinal autonomic function in support of a diagnosis.

Diagnostic tests of cardiac autonomic neuropathy may serve as a surrogate for autonomic neuropathy within the GI system, but associations between autonomic neuropathy in the two different visceral systems remain incompletely understood. Reduced heart rate variability is associated with hyposensitivity of the esophagus and with hyposensitivity and stretch of the rectum in patients with DM [34,124,158]. However, results are ambiguous regarding associations between GI transit times and cardiac derived autonomic parameters such as heart rate variability or cardiac vagal tone [46]. Cardiac parasympathetic dysfunction can be verified by demonstrating decreased heart rate variability during rest, deep breathing, and the Valsalva maneuver [159]. Heart rate changes to deep breathing are simple to perform and have the highest specificity with vagal afferents and efferents mediating the response [160]. The efferent cardiovascular adrenergic function can be assessed by looking at blood pressure changes during the Valsalva maneuver, during orthostatic stress (active standing or tilt table testing), in response to isometric exercise, and a cold pressor test [161,162,163]. Twenty-four hour blood pressure measurement may detect non-dipping or reverse dipping conditions and postprandial hypotension. The prognostic role of non-dipping and reverse dipping is well-documented, but associations with GI function are unknown [164,165].

Further autonomic testing may be relevant in some patients to recognize AD. A commonly used questionnaire is the COMPASS-31, consisting of 31 questions formed into six symptom domains [38], which may be helpful to screen for AD-related symptoms and add to the assessment of GI autonomic impairment. Autonomic symptoms reflect the organ or function that is affected; however, in general, they are unspecific and will often require objective assessment with various tests [20,166,167,168]. Additionally, *The Quantitative Sweat Measurement System* (Q-Sweat) evaluates the postganglionic sympathetic cholinergic sudomotor function in the upper and lower extremities by measuring sweat collections in response to locally administered acetylcholine [169]. In combination with skin biopsies and quantitative sensory testing, the Q-Sweat contributes to the diagnosis of small-fiber polyneuropathy [14]. Normal values are based on published normative data [160]. Serum pancreatic polypeptide is an indirect measure of vagal influence on the GI tract [170,171], but its utility remains to be determined [172].

With no available standard diagnostic test of pan-enteric autonomic neuropathy, extraintestinal autonomic neuropathy may be used as proxy in clinical practice to verify AD outside the GI tract. However, acknowledgement of subjective GI symptoms and assessment of the physiological function of each GI segment remains the primary focus to aid diagnostics and guide treatment in patients with GI symptoms and suspected AD.

## 6. Assessment-Guided Treatment

Management of AD-related gastroenteropathy is challenging and treatment response is often unsatisfactory. The poor correlation between GI symptoms and objective findings underlines the need for objective measures to guide treatment.

The risk of malnutrition, electrolyte disturbances, weight loss, and dehydration is increased in patients with gastroparesis and enteropathy. Small and soft meals, preferably low in fat and fiber content, are recommended to ease gastric emptying and optimize the intestinal nutritional uptake in patients with gastroparesis or constipation [173]. Contrary, an increased fiber intake is shown to reduce symptoms of constipation and optimize medication absorption in PD [174]. To preserve a sufficient nutritional state, a feeding tube may be necessary for selected patients with weight loss.

Improvement of glycemic control and variability is important in patients with DM to reduce the risk of dysmotility due to hyper- or hypoglycemia. Continuous subcutaneous insulin infusion, and by this an optimized glycemic regulation, may diminish GI symptoms; however, the magnitude of this effect is uncertain [175].

Pharmacologic treatment with prokinetic drugs is widely used when upper GI symptoms combined with an objectively measured delayed GE or prolonged intestinal transit times are detected. The dopamine receptor antagonists (metoclopramide and domperidone), the motilin receptor agonist (erythromycin), and the selective 5-HT_4_ receptor agonist (prucalopride) are commonly used in clinical practice [176]. However, prokinetic treatment has major limitations, especially the risk of extrapyramidal side effects including potentially irreversible tardive dyskinesia (metoclopramide), drug-induced arrhythmias (domperidone), and lack of evidence for long-term effectiveness [177,178]. In addition, metoclopramide is contraindicated in PD due to its extrapyramidal side effects [24]. Ghrelin receptor agonists (relamorelin and ulimorelin) may be a future treatment of gastroparesis, but solid evidence remains absent [175]. Immunotherapy in autoimmune autonomic ganglionopathy which can comprise gastroparesis is well indicated [179].

In medical refractory cases of gastroparesis, gastric electrical stimulation is the most used surgical option, but disagreement in randomized studies remains. Especially in diabetic gastroparesis, studies have shown significant symptom relief, maintained for over 10 years, and a reduction in days of hospitalization [180,181]. Other surgical interventions used for treating dysmotility within the upper GI tract comprise pyloric botulinum toxin injection, pyloroplasty, pyloromyotomy, gastrectomy, and gastric per-oral endoscopic myotomy (G-POEM). In general, surgical interventions rest on poor evidence, and patients should be carefully selected. A non-invasive neuromodulation technique, called transcutaneous vagus nerve stimulation, is investigated as a potential add-on treatment of GI symptoms in patients with DM and AD [182].

When SIBO is objectively verified, patients are treated with antibiotics to eradicate bacterial overgrowth, which provides significant symptom relief and enhances medication absorption. Either non-systemic antibiotics (rifaximin) or systemic antibiotics (ciprofloxacin or metronidazole) can be used [32]. However, the predisposing motility disturbance causes frequent recurrence [183]. The anti-diarrheal, peripherally acting u-opioid receptor agonist, loperamide, reduces intestinal peristalsis and can be effective in treating diarrhea and fecal incontinence. Moreover, octreotide, ondansetron, and bile-binding resins are used in selected patients with severe diarrhea [184]. When paralytic ileus occurs, neostigmine may be used in selected cases.

Prolonged colonic transit time indicates treatment with oral laxatives or suppositories following the general guidelines of treating chronic constipation [24]. If ordinary treatment fails, this may be combined with prucalopride due to its additional prokinetic effects [176]. When constipation coexists with abdominal pain and autonomic neuropathy, simple and adjuvant analgesics such as tricyclic antidepressants may be attempted. The balance between the relatively low analgesic effect and the frequent side effects must always be considered. The cholinesterase-inhibitor pyridostigmine is frequently used in patients with combined orthostatic hypotension and constipation [18]. Patients with comorbid mast cell activation syndrome may achieve symptomatic improvement when treated with mast cell stabilizers, such as anti-histamine and cromolyn sodium [16].

When obstructed defecation is verified with anorectal manometry, it is usually treated with rectal suppositories or mini-enema. Confirmed dyssynergic defecation in PD may be treated with injections of botulinum neurotoxin [24].

## 7. Conclusions

Pan-enteric dysmotility is common in patients with AD despite variation in the underlying pathophysiological changes within the nervous system. With no available standard method for direct assessment of GI autonomic neuropathy, the primary diagnostic approach is physiological, multi-segmental motility testing, and in some patients additional generalized tests of autonomic neuropathy.

Established assessment methods are commercially available for investigation of transit times throughout the entire GI tract and for contraction patterns in the esophageal, gastroduodenal, and anal regions. As the only commercially available method, the wireless motility capsule provides pan-enteric transit times and pressure patterns in one investigation. However, the established methods all present limitations, especially with regards to radiation exposure, lack of standardization, need for multiple tests to evaluate the entire GI tract, and a complicated practical performance or data interpretation, which may restrict the use to specialized centers.

Within recent years, several emerging assessment methods have been developed, potentially overcoming some of the above limitations and definitely providing more detailed knowledge on contractility patterns within specific GI segments. The 3D-Transit system, CT scans, and MRI scans hold promise for a multi-segmental and detailed evaluation of the whole GI tract within a single investigation. In the future, the diagnosis of enteric autonomic neuropathy may be established with ^11^C-donepezil PET/CT scans or gut biopsies. Optimized future diagnostic tools and improved knowledge on motility disturbances in gastroenteropathy related to AD will hopefully improve the treatment of these severely ill patients.

## Figures and Tables

**Table 1:** Established and emerging methods for assessment of gastroenteropathy in autonomic disorders.

Investigation	Measurement	Primary Dysmotility Parameters	Advantages/Limitations							
			Minimally invasive	Radiation free	Standardized	Inexpensive	High Availability	Ambulatory assessment	Simple data analysis	High reliability
**Established Assessment Methods**										
Esophageal manometry	Esophageal contractility patterns	Reduced peristalsis and uncoordinated contractions	No	Yes	Yes	No	No	No	No	Yes
Gastric emptying scintigraphy	Gastric emptying time	Delayed gastric emptying time	Yes	No	Yes	No	Yes	No	No	Yes
^13^C-octanoic acid breath test	Gastric emptying time	Delayed gastric emptying time	Yes	Yes	Yes	Yes	No	No	Yes	No
Antropyloroduodenal manometry	Antropyloroduodenal contractility patterns	Postprandial antral hypomotility and duodenal dysmotility in diabetes	No	Yes	No	No	No	No/Yes	No	Yes
Intestinal scintigraphy	Small intestinal and colonic transit times	Prolonged intestinal transit times	Yes	No	No	No	No	No	No	Yes
Radio-opaque markers	Small intestinal and colonic transit times	Prolonged whole gut and regional transit times	Yes	No	No	Yes	Yes	Yes	Yes	No
Hydrogen and methane breath test	Orocecal transit time and detection of small intestinal bacterial overgrowth	Prolonged orocecal transit time and increased frequency of small intestinal bacterial overgrowth	Yes	Yes	Yes	Yes	Yes	No	Yes	No
Anorectal manometry	Anorectal contractility patterns	1. Dystonic external anal sphincter during defecation in Parkinson’s disease 2. Dysfunction of the internal anal sphincter in diabetes 3. Recto-anal dyscoordination	Yes	Yes	No	No	No	No	No	Yes
Wireless motility capsule	1. Whole gut and regional transit times 2. Motility patterns	Delayed whole gut- and regional transit times	Yes	Yes	Yes	No	No	Yes	Yes	Yes
**Emerging Assessment Methods**										
Colonic Manometry	Colonic contractility patterns	Colonic dysmotility	No	Yes	No	No	No	No	No	Yes
3D-Transit capsule	Whole gut and regional transit times	Delayed whole gut- and regional transit times	Yes	Yes	No	No	No	Yes	No	Yes
Computed tomography imaging	Small intestinal and colonic volume	Increased colonic volume	Yes	No	No	Yes	Yes	No	No	Yes
Magnetic resonance imaging	1. Whole gut and regional transit times 2. Whole gut contractility 3. Organ volumes	Delayed gastric emptying and increased intestinal volume	Yes	Yes	No	No	Yes	No	No	Yes
^11^C-donepezil positron emission tomography/computed tomography imaging	Whole gut cholinergic innervation	Intestinal parasympathetic denervation	Yes	No	No	No	No	No	No	Yes
Submucosal biopsies	Quantification of enteric neurons	1. Reduced number of neurons in diabetes 2. Aggregation of α-synuclein in Parkinson’s disease	No	Yes	No	No	No	No	No	No
Microbiota	Gut microbiota composition	1. Less stable and diverse in diabetes 2. Altered in Parkinson’s disease	Yes	Yes	No	No	No	Yes	No	No

## References

[1] Benarroch E.E. (2020). Physiology and Pathophysiology of the Autonomic Nervous System. Continuum.

[2] Mathias C.J.B.R. (2013). Autonomic Failure. A Textbook of Clinical Disorders of the Autonomic Nervous System.

[3] Chung K.A., Pfeiffer R.F. (2020). Gastrointestinal dysfunction in the synucleinopathies. Clin. Auton. Res..

[4] Müller T., Erdmann C., Bremen D., Schmidt W.E., Muhlack S., Woitalla D., Goetze O. (2006). Impact of gastric emptying on levodopa pharmacokinetics in Parkinson disease patients. Clin. Neuropharmacol..

[5] Knudsen K., Fedorova T.D., Bekker A.C., Iversen P., Østergaard K., Krogh K., Borghammer P. (2017). Objective Colonic Dysfunction is Far more Prevalent than Subjective Constipation in Parkinson’s Disease: A Colon Transit and Volume Study. J. Park. Dis..

[6] Rouphael C., Arora Z., Thota P.N., Lopez R., Santisi J., Funk C., Cline M. (2017). Role of wireless motility capsule in the assessment and management of gastrointestinal dysmotility in patients with diabetes mellitus. Neurogastroenterol. Motil..

[7] Gustafsson R.J., Littorin B., Berntorp K., Frid A., Thorsson O., Olsson R., Ekberg O., Ohlsson B. (2011). Esophageal dysmotility is more common than gastroparesis in diabetes mellitus and is associated with retinopathy. Rev. Diabet. Stud..

[8] Bharucha A.E., Camilleri M., Forstrom L.A., Zinsmeister A.R. (2009). Relationship between clinical features and gastric emptying disturbances in diabetes mellitus. Clin. Endocrinol..

[9] Karemaker J.M. (2017). An introduction into autonomic nervous function. Physiol. Meas..

[10] Costa M., Brookes S.J. (1994). The enteric nervous system. Am. J. Gastroenterol..

[11] Meldgaard T., Olesen S.S., Farmer A.D., Krogh K., Wendel A.A., Brock B., Drewes A.M., Brock C. (2018). Diabetic Enteropathy: From Molecule to Mechanism-Based Treatment. J. Diabetes Res..

[12] Gibbons C.H., Freeman R. (2015). Treatment-induced neuropathy of diabetes: An acute, iatrogenic complication of diabetes. Brain.

[13] Coon E.A., Cutsforth-Gregory J.K., Benarroch E.E. (2018). Neuropathology of autonomic dysfunction in synucleinopathies. Mov. Disord..

[14] Terkelsen A.J., Karlsson P., Lauria G., Freeman R., Finnerup N.B., Jensen T.S. (2017). The diagnostic challenge of small fibre neuropathy: Clinical presentations, evaluations, and causes. Lancet Neurol..

[15] Benarroch E.E. (2012). Postural tachycardia syndrome: A heterogeneous and multifactorial disorder. Mayo Clin. Proc..

[16] Weinstock L.B., Pace L.A., Rezaie A., Afrin L.B., Molderings G.J. (2020). Mast Cell Activation Syndrome: A Primer for the Gastroenterologist. Dig. Dis. Sci..

[17] Beckers A.B., Keszthelyi D., Fikree A., Vork L., Masclee A., Farmer A.D., Aziz Q. (2017). Gastrointestinal disorders in joint hypermobility syndrome/Ehlers-Danlos syndrome hypermobility type: A review for the gastroenterologist. Neurogastroenterol. Motil..

[18] Benarroch E.E. (2014). The clinical approach to autonomic failure in neurological disorders. Nat. Rev. Neurol..

[19] Soedamah-Muthu S.S., Chaturvedi N., Witte D.R., Stevens L.K., Porta M., Fuller J.H. (2008). Relationship between risk factors and mortality in type 1 diabetic patients in Europe: The EURODIAB Prospective Complications Study (PCS). Diabetes Care.

[20] Spallone V. (2019). Update on the Impact, Diagnosis and Management of Cardiovascular Autonomic Neuropathy in Diabetes: What Is Defined, What Is New, and What Is Unmet. Diabetes Metab J..

[21] Bharucha A.E., Kudva Y.C., Prichard D.O. (2019). Diabetic Gastroparesis. Endocr. Rev..

[22] Bytzer P., Talley N.J., Leemon M., Young L.J., Jones M.P., Horowitz M. (2001). Prevalence of gastrointestinal symptoms associated with diabetes mellitus: A population-based survey of 15,000 adults. Arch. Int. Med..

[23] Talley N.J., Young L., Bytzer P., Hammer J., Leemon M., Jones M., Horowitz M. (2001). Impact of chronic gastrointestinal symptoms in diabetes mellitus on health-related quality of life. Am. J. Gastroenterol..

[24] Fasano A., Visanji N.P., Liu L.W., Lang A.E., Pfeiffer R.F. (2015). Gastrointestinal dysfunction in Parkinson’s disease. Lancet Neurol..

[25] Bytzer P., Talley N.J., Hammer J., Young L.J., Jones M.P., Horowitz M. (2002). GI symptoms in diabetes mellitus are associated with both poor glycemic control and diabetic complications. Am. J. Gastroenterol..

[26] Feldman M., Schiller L.R. (1983). Disorders of gastrointestinal motility associated with diabetes mellitus. Ann. Intern. Med..

[27] Knudsen K., Krogh K., Østergaard K., Borghammer P. (2017). Constipation in parkinson’s disease: Subjective symptoms, objective markers, and new perspectives. Mov. Disord..

[28] Verbaan D., Marinus J., Visser M., van Rooden S.M., Stiggelbout A.M., van Hilten J.J. (2007). Patient-reported autonomic symptoms in Parkinson disease. Neurology.

[29] Wang L.B., Culbertson C.J., Deb A., Morgenshtern K., Huang H., Hohler A.D. (2015). Gastrointestinal dysfunction in postural tachycardia syndrome. J. Neurol. Sci..

[30] DiBaise J.K., Harris L.A., Goodman B. (2018). Postural Tachycardia Syndrome (POTS) and the GI Tract: A Primer for the Gastroenterologist. Am. J. Gastroenterol..

[31] Claus I., Suttrup J., Muhle P., Suntrup-Krueger S., Siemer M.L., Lenze F., Dziewas R., Warnecke T. (2018). Subtle Esophageal Motility Alterations in Parkinsonian Syndromes: Synucleinopathies vs. Tauopathies. Mov. Disord. Clin. Pract..

[32] Rao S.S.C., Bhagatwala J. (2019). Small Intestinal Bacterial Overgrowth: Clinical Features and Therapeutic Management. Clin. Transl. Gastroenterol..

[33] Knudsen K., Borghammer P. (2018). Imaging the Autonomic Nervous System in Parkinson’s Disease. Curr. Neurol. Neurosci. Rep..

[34] Brock C., Søfteland E., Gunterberg V., Frøkjær J.B., Lelic D., Brock B., Dimcevski G., Gregersen H., Simrén M., Drewes A.M. (2013). Diabetic autonomic neuropathy affects symptom generation and brain-gut axis. Diabetes Care.

[35] Azpiroz F., Malagelada C. (2016). Diabetic neuropathy in the gut: Pathogenesis and diagnosis. Diabetologia.

[36] Farmer A.D., Pedersen A.G., Brock B., Jakobsen P.E., Karmisholt J., Mohammed S.D., Scott S.M., Drewes A.M., Brock C. (2017). Type 1 diabetic patients with peripheral neuropathy have pan-enteric prolongation of gastrointestinal transit times and an altered caecal pH profile. Diabetologia.

[37] Pavelić A., Krbot Skorić M., Crnošija L., Habek M. (2017). Postprandial hypotension in neurological disorders: Systematic review and meta-analysis. Clin. Auton. Res..

[38] Sletten D.M., Suarez G.A., Low P.A., Mandrekar J., Singer W. (2012). COMPASS 31: A refined and abbreviated Composite Autonomic Symptom Score. Mayo Clin. Proc..

[39] Rentz A.M., Kahrilas P., Stanghellini V., Tack J., Talley N.J., de la Loge C., Trudeau E., Dubois D., Revicki D.A. (2004). Development and psychometric evaluation of the patient assessment of upper gastrointestinal symptom severity index (PAGI-SYM) in patients with upper gastrointestinal disorders. Qual. Life Res..

[40] Revicki D.A., Rentz A.M., Dubois D., Kahrilas P., Stanghellini V., Talley N.J., Tack J. (2004). Gastroparesis Cardinal Symptom Index (GCSI): Development and validation of a patient reported assessment of severity of gastroparesis symptoms. Qual. Life Res. Int. J. Qual. Life Asp. Treat. Care Rehabil..

[41] Nilsson M., Poulsen J.L., Brock C., Sandberg T.H., Gram M., Frokjaer J.B., Krogh K., Drewes A.M. (2016). Opioid-induced bowel dysfunction in healthy volunteers assessed with questionnaires and MRI. Eur. J. Gastroenterol. Hepatol..

[42] Jones K.L., Russo A., Stevens J.E., Wishart J.M., Berry M.K., Horowitz M. (2001). Predictors of delayed gastric emptying in diabetes. Diabetes Care.

[43] Cassilly D.W., Wang Y.R., Friedenberg F.K., Nelson D.B., Maurer A.H., Parkman H.P. (2008). Symptoms of gastroparesis: Use of the gastroparesis cardinal symptom index in symptomatic patients referred for gastric emptying scintigraphy. Digestion.

[44] Kulich K.R., Madisch A., Pacini F., Piqué J.M., Regula J., Van Rensburg C.J., Ujszászy L., Carlsson J., Halling K., Wiklund I.K. (2008). Reliability and validity of the Gastrointestinal Symptom Rating Scale (GSRS) and Quality of Life in Reflux and Dyspepsia (QOLRAD) questionnaire in dyspepsia: A six-country study. Health Qual. Life Outcomes.

[45] Revicki D.A., Wood M., Wiklund I., Crawley J. (1998). Reliability and validity of the Gastrointestinal Symptom Rating Scale in patients with gastroesophageal reflux disease. Qual. Life Res..

[46] Wegeberg A.L., Brock C., Ejskjaer N., Karmisholt J.S., Jakobsen P.E., Drewes A.M., Brock B., Farmer A.D. (2020). Gastrointestinal symptoms and cardiac vagal tone in type 1 diabetes correlates with gut transit times and motility index. Neurogastroenterol. Motil..

[47] Agachan F., Chen T., Pfeifer J., Reissman P., Wexner S.D. (1996). A constipation scoring system to simplify evaluation and management of constipated patients. Dis. Colon Rectum.

[48] Simren M., Palsson O.S., Whitehead W.E. (2017). Update on Rome IV Criteria for Colorectal Disorders: Implications for Clinical Practice. Curr. Gastroenterol. Rep..

[49] Quan C., Talley N.J., Cross S., Jones M., Hammer J., Giles N., Horowitz M. (2003). Development and validation of the Diabetes Bowel Symptom Questionnaire. Aliment. Pharmacol. Ther..

[50] Kahrilas P.J., Bredenoord A.J., Fox M., Gyawali C.P., Roman S., Smout A.J., Pandolfino J.E. (2015). The Chicago Classification of esophageal motility disorders, v3.0. Neurogastroenterol. Motil..

[51] Dhawan I., O’Connell B., Patel A., Schey R., Parkman H.P., Friedenberg F. (2018). Utility of Esophageal High-Resolution Manometry in Clinical Practice: First, Do HRM. Dig. Dis. Sci..

[52] Yadlapati R. (2017). High-resolution esophageal manometry: Interpretation in clinical practice. Curr. Opin. Gastroenterol..

[53] George N.S., Rangan V., Geng Z., Khan F., Kichler A., Gabbard S., Ganocy S., Fass R. (2017). Distribution of Esophageal Motor Disorders in Diabetic Patients With Dysphagia. J. Clin. Gastroenterol..

[54] Huang R.J., Chun C.L., Friday K., Triadafilopoulos G. (2013). Manometric abnormalities in the postural orthostatic tachycardia syndrome: A case series. Dig. Dis. Sci..

[55] Fikree A., Aziz Q., Sifrim D. (2017). Mechanisms underlying reflux symptoms and dysphagia in patients with joint hypermobility syndrome, with and without postural tachycardia syndrome. Neurogastroenterol. Motil..

[56] Martin-Harris B., Canon C.L., Bonilha H.S., Murray J., Davidson K., Lefton-Greif M.A. (2020). Best Practices in Modified Barium Swallow Studies. Am. J. Speech Lang. Pathol..

[57] Schiffer B.L., Kendall K. (2019). Changes in Timing of Swallow Events in Parkinson’s Disease. Ann. Otol. Rhinol. Laryngol..

[58] Lee J.W., Randall D.R., Evangelista L.M., Kuhn M.A., Belafsky P.C. (2017). Subjective Assessment of Videofluoroscopic Swallow Studies. Otolaryngol. Head Neck Surg..

[59] Alomari M., Hitawala A., Chadalavada P., Covut F., Al Momani L., Khazaaleh S., Gosai F., Al Ashi S., Abushahin A., Schneider A. (2020). Prevalence and Predictors of Gastrointestinal Dysmotility in Patients with Hypermobile Ehlers-Danlos Syndrome: A Tertiary Care Center Experience. Cureus.

[60] Abell T.L., Camilleri M., Donohoe K., Hasler W.L., Lin H.C., Maurer A.H., McCallum R.W., Nowak T., Nusynowitz M.L., Parkman H.P. (2008). Consensus recommendations for gastric emptying scintigraphy: A joint report of the American Neurogastroenterology and Motility Society and the Society of Nuclear Medicine. Am. J. Gastroenterol..

[61] Rao S.S., Camilleri M., Hasler W.L., Maurer A.H., Parkman H.P., Saad R., Scott M.S., Simren M., Soffer E., Szarka L. (2011). Evaluation of gastrointestinal transit in clinical practice: Position paper of the American and European Neurogastroenterology and Motility Societies. Neurogastroenterol. Motil..

[62] Madsen J.L. (2014). Scintigraphic assessment of gastrointestinal motility: A brief review of techniques and data interpretation. Clin. Physiol. Funct. Imaging.

[63] Thomaides T., Karapanayiotides T., Zoukos Y., Haeropoulos C., Kerezoudi E., Demacopoulos N., Floodas G., Papageorgiou E., Armakola F., Thomopoulos Y. (2005). Gastric emptying after semi-solid food in multiple system atrophy and Parkinson disease. J. Neurol..

[64] Loavenbruck A., Iturrino J., Singer W., Sletten D.M., Low P.A., Zinsmeister A.R., Bharucha A.E. (2015). Disturbances of gastrointestinal transit and autonomic functions in postural orthostatic tachycardia syndrome. Neurogastroenterol. Motil..

[65] Ghoos Y.F., Maes B.D., Geypens B.J., Mys G., Hiele M.I., Rutgeerts P.J., Vantrappen G. (1993). Measurement of gastric emptying rate of solids by means of a carbon-labeled octanoic acid breath test. Gastroenterology.

[66] Zahn A., Langhans C.D., Hoffner S., Haberkorn U., Rating D., Haass M., Enck P., Stremmel W., Ruhl A. (2003). Measurement of gastric emptying by 13C-octanoic acid breath test versus scintigraphy in diabetics. Z. Gastroenterol..

[67] Tanaka Y., Kato T., Nishida H., Yamada M., Koumura A., Sakurai T., Hayashi Y., Kimura A., Hozumi I., Araki H. (2012). Is there delayed gastric emptying in patients with multiple system atrophy? An analysis using the (13)C-acetate breath test. J. Neurol..

[68] Knudsen K., Szwebs M., Hansen A.K., Borghammer P. (2018). Gastric emptying in Parkinson’s disease-A mini-review. Parkinsonism. Relat. Disord..

[69] Camilleri M., Bharucha A.E., di Lorenzo C., Hasler W.L., Prather C.M., Rao S.S., Wald A. (2008). American Neurogastroenterology and Motility Society consensus statement on intraluminal measurement of gastrointestinal and colonic motility in clinical practice. Neurogastroenterol. Motil..

[70] Samsom M., Jebbink R.J., Akkermans L.M., van Berge-Henegouwen G.P., Smout A.J. (1996). Abnormalities of antroduodenal motility in type I diabetes. Diabetes Care.

[71] Patcharatrakul T., Gonlachanvit S. (2013). Technique of functional and motility test: How to perform antroduodenal manometry. J. Neurogastroenterol. Motil..

[72] Penning C., Gielkens H.A., Hemelaar M., Lamers C.B., Masclee A.A. (2001). Reproducibility of antroduodenal motility during prolonged ambulatory recording. Neurogastroenterol. Motil..

[73] Bortolotti M., Annese V., Coccia G. (2000). Twenty-four hour ambulatory antroduodenal manometry in normal subjects (co-operative study). Neurogastroenterol. Motil..

[74] Desipio J., Friedenberg F.K., Korimilli A., Richter J.E., Parkman H.P., Fisher R.S. (2007). High-resolution solid-state manometry of the antropyloroduodenal region. Neurogastroenterol. Motil..

[75] Bonapace E.S., Maurer A.H., Davidoff S., Krevsky B., Fisher R.S., Parkman H.P. (2000). Whole gut transit scintigraphy in the clinical evaluation of patients with upper and lower gastrointestinal symptoms. Am. J. Gastroenterol..

[76] Maleki D., Camilleri M., Burton D.D., Rath-Harvey D.M., Oenning L., Pemberton J.H., Low P.A. (1998). Pilot study of pathophysiology of constipation among community diabetics. Dig. Dis. Sci..

[77] Camilleri M., Thorne N.K., Ringel Y., Hasler W.L., Kuo B., Esfandyari T., Gupta A., Scott S.M., McCallum R.W., Parkman H.P. (2010). Wireless pH-motility capsule for colonic transit: Prospective comparison with radiopaque markers in chronic constipation. Neurogastroenterol. Motil..

[78] van der Sijp J.R., Kamm M.A., Nightingale J.M., Britton K.E., Mather S.J., Morris G.P., Akkermans L.M., Lennard-Jones J.E. (1993). Radioisotope determination of regional colonic transit in severe constipation: Comparison with radio opaque markers. Gut.

[79] Abrahamsson H., Antov S., Bosaeus I. (1988). Gastrointestinal and colonic segmental transit time evaluated by a single abdominal X-ray in healthy subjects and constipated patients. Scand. J. Gastroenterol. Suppl..

[80] Metcalf A.M., Phillips S.F., Zinsmeister A.R., MacCarty R.L., Beart R.W., Wolff B.G. (1987). Simplified assessment of segmental colonic transit. Gastroenterology.

[81] Sakakibara R., Odaka T., Uchiyama T., Liu R., Asahina M., Yamaguchi K., Yamaguchi T., Yamanishi T., Hattori T. (2004). Colonic transit time, sphincter EMG, and rectoanal videomanometry in multiple system atrophy. Mov. Disord..

[82] Jung H.K., Kim D.Y., Moon I.H., Hong Y.S. (2003). Colonic transit time in diabetic patients--comparison with healthy subjects and the effect of autonomic neuropathy. Yonsei Med. J..

[83] Miller M.A., Parkman H.P., Urbain J.L., Brown K.L., Donahue D.J., Knight L.C., Maurer A.H., Fisher R.S. (1997). Comparison of scintigraphy and lactulose breath hydrogen test for assessment of orocecal transit: Lactulose accelerates small bowel transit. Dig. Dis. Sci..

[84] Simrén M., Stotzer P.O. (2006). Use and abuse of hydrogen breath tests. Gut.

[85] Faria M., Pavin E.J., Parisi M.C., Lorena S.L., Brunetto S.Q., Ramos C.D., Pavan C.R., Mesquita M.A. (2013). Delayed small intestinal transit in patients with long-standing type 1 diabetes mellitus: Investigation of the relationships with clinical features, gastric emptying, psychological distress, and nutritional parameters. Diabetes Technol. Ther..

[86] Davies K.N., King D., Billington D., Barrett J.A. (1996). Intestinal permeability and orocaecal transit time in elderly patients with Parkinson’s disease. Postgrad. Med. J..

[87] Gabrielli M., Bonazzi P., Scarpellini E., Bendia E., Lauritano E.C., Fasano A., Ceravolo M.G., Capecci M., Rita Bentivoglio A., Provinciali L. (2011). Prevalence of small intestinal bacterial overgrowth in Parkinson’s disease. Mov. Disord..

[88] Jacobs C., Coss Adame E., Attaluri A., Valestin J., Rao S.S. (2013). Dysmotility and proton pump inhibitor use are independent risk factors for small intestinal bacterial and/or fungal overgrowth. Aliment. Pharmacol. Ther..

[89] Rezaie A., Buresi M., Lembo A., Lin H., McCallum R., Rao S., Schmulson M., Valdovinos M., Zakko S., Pimentel M. (2017). Hydrogen and Methane-Based Breath Testing in Gastrointestinal Disorders: The North American Consensus. Am. J. Gastroenterol..

[90] Di Stefano M., Quigley E.M.M. (2018). The diagnosis of small intestinal bacterial overgrowth: Two steps forward, one step backwards?. Neurogastroenterol. Motil..

[91] Yu D., Cheeseman F., Vanner S. (2011). Combined oro-caecal scintigraphy and lactulose hydrogen breath testing demonstrate that breath testing detects oro-caecal transit, not small intestinal bacterial overgrowth in patients with IBS. Gut.

[92] Scott S.M., Carrington E.V. (2020). The London Classification: Improving Characterization and Classification of Anorectal Function with Anorectal Manometry. Curr. Gastroenterol. Rep..

[93] Carrington E.V., Heinrich H., Knowles C.H., Fox M., Rao S., Altomare D.F., Bharucha A.E., Burgell R., Chey W.D., Chiarioni G. (2020). The international anorectal physiology working group (IAPWG) recommendations: Standardized testing protocol and the London classification for disorders of anorectal function. Neurogastroenterol. Motil..

[94] Lee T.H., Bharucha A.E. (2016). How to Perform and Interpret a High-resolution Anorectal Manometry Test. J. Neurogastroenterol. Motil..

[95] Oblizajek N.R., Gandhi S., Sharma M., Chakraborty S., Muthyala A., Prichard D., Feuerhak K., Bharucha A.E. (2019). Anorectal pressures measured with high-resolution manometry in healthy people-Normal values and asymptomatic pelvic floor dysfunction. Neurogastroenterol. Motil..

[96] Li Y., Yang X., Xu C., Zhang Y., Zhang X. (2013). Normal values and pressure morphology for three-dimensional high-resolution anorectal manometry of asymptomatic adults: A study in 110 subjects. Int. J. Colorectal Dis..

[97] De Pablo-Fernández E., Passananti V., Zárate-López N., Emmanuel A., Warner T. (2019). Colonic transit, high-resolution anorectal manometry and MRI defecography study of constipation in Parkinson’s disease. Parkinsonism Relat. Disord..

[98] Yu T., Wang Y., Wu G., Xu Q., Tang Y., Lin L. (2016). High-resolution Anorectal Manometry in Parkinson Disease With Defecation Disorder: A Comparison With Functional Defecation Disorder. J. Clin. Gastroenterol..

[99] Sarosiek I., Selover K.H., Katz L.A., Semler J.R., Wilding G.E., Lackner J.M., Sitrin M.D., Kuo B., Chey W.D., Hasler W.L. (2010). The assessment of regional gut transit times in healthy controls and patients with gastroparesis using wireless motility technology. Aliment. Pharmacol. Ther..

[100] Wang Y.T., Mohammed S.D., Farmer A.D., Wang D., Zarate N., Hobson A.R., Hellstrom P.M., Semler J.R., Kuo B., Rao S.S. (2015). Regional gastrointestinal transit and pH studied in 215 healthy volunteers using the wireless motility capsule: Influence of age, gender, study country and testing protocol. Aliment. Pharmacol. Ther..

[101] Farmer A.D., Wegeberg A.L., Brock B., Hobson A.R., Mohammed S.D., Scott S.M., Bruckner-Holt C.E., Semler J.R., Hasler W.L., Hellstrom P.M. (2018). Regional gastrointestinal contractility parameters using the wireless motility capsule: Inter-observer reproducibility and influence of age, gender and study country. Aliment. Pharmacol. Ther..

[102] Maqbool S., Parkman H.P., Friedenberg F.K. (2009). Wireless capsule motility: Comparison of the SmartPill GI monitoring system with scintigraphy for measuring whole gut transit. Dig. Dis. Sci..

[103] Kuo B., McCallum R.W., Koch K.L., Sitrin M.D., Wo J.M., Chey W.D., Hasler W.L., Lackner J.M., Katz L.A., Semler J.R. (2008). Comparison of gastric emptying of a nondigestible capsule to a radio-labelled meal in healthy and gastroparetic subjects. Aliment. Pharmacol. Ther..

[104] Rao S.S., Kuo B., McCallum R.W., Chey W.D., DiBaise J.K., Hasler W.L., Koch K.L., Lackner J.M., Miller C., Saad R. (2009). Investigation of colonic and whole-gut transit with wireless motility capsule and radiopaque markers in constipation. Clin. Gastroenterol. Hepatol..

[105] Fraser R.J., Horowitz M., Maddox A.F., Harding P.E., Chatterton B.E., Dent J. (1990). Hyperglycaemia slows gastric emptying in type 1 (insulin-dependent) diabetes mellitus. Diabetologia.

[106] Zhou W., Zikos T.A., Clarke J.O., Nguyen L.A., Triadafilopoulos G., Neshatian L. (2021). Regional Gastrointestinal Transit and Contractility Patterns Vary in Postural Orthostatic Tachycardia Syndrome (POTS). Dig. Dis. Sci..

[107] Su A., Gandhy R., Barlow C., Triadafilopoulos G. (2017). Utility of the wireless motility capsule and lactulose breath testing in the evaluation of patients with Parkinson’s disease who present with functional gastrointestinal symptoms. BMJ Open Gastroenterol..

[108] Coleski R., Wilding G.E., Semler J.R., Hasler W.L. (2015). Blunting of Colon Contractions in Diabetics with Gastroparesis Quantified by Wireless Motility Capsule Methods. PLoS ONE.

[109] Mark E.B., Poulsen J.L., Haase A.M., Espersen M., Gregersen T., Schlageter V., Scott S.M., Krogh K., Drewes A.M. (2019). Ambulatory assessment of colonic motility using the electromagnetic capsule tracking system. Neurogastroenterol. Motil..

[110] Haase A.M., Gregersen T., Schlageter V., Scott M.S., Demierre M., Kucera P., Dahlerup J.F., Krogh K. (2014). Pilot study trialling a new ambulatory method for the clinical assessment of regional gastrointestinal transit using multiple electromagnetic capsules. Neurogastroenterol. Motil..

[111] Worsoe J., Fynne L., Gregersen T., Schlageter V., Christensen L.A., Dahlerup J.F., Rijkhoff N.J., Laurberg S., Krogh K. (2011). Gastric transit and small intestinal transit time and motility assessed by a magnet tracking system. BMC Gastroenterol..

[112] Brinck C.E., Mark E.B., Winther Klinge M., Ejerskov C., Sutter N., Schlageter V., Scott S.M., Mohr Drewes A., Krogh K. (2020). Magnetic tracking of gastrointestinal motility. Physiol. Meas..

[113] Sutter N., Klinge M.W., Mark E.B., Nandhra G., Haase A.M., Poulsen J., Knudsen K., Borghammer P., Schlageter V., Birch M. (2020). Normative values for gastric motility assessed with the 3D-transit electromagnetic tracking system. Neurogastroenterol. Motil..

[114] Nandhra G.K., Mark E.B., Di Tanna G.L., Haase A.M., Poulsen J., Christodoulides S., Kung V., Klinge M.W., Knudsen K., Borghammer P. (2020). Normative values for region-specific colonic and gastrointestinal transit times in 111 healthy volunteers using the 3D-Transit electromagnet tracking system: Influence of age, gender, and body mass index. Neurogastroenterol. Motil..

[115] Klinge M.W., Haase A.M., Mark E.B., Sutter N., Fynne L.V., Drewes A.M., Schlageter V., Lund S., Borghammer P., Krogh K. (2020). Colonic motility in patients with type 1 diabetes and gastrointestinal symptoms. Neurogastroenterol. Motil..

[116] Knudsen K., Haase A.M., Fedorova T.D., Bekker A.C., Ostergaard K., Krogh K., Borghammer P. (2017). Gastrointestinal Transit Time in Parkinson’s Disease Using a Magnetic Tracking System. J. Park. Dis..

[117] Dinning P.G., Carrington E.V., Scott S.M. (2015). The use of colonic and anorectal high-resolution manometry and its place in clinical work and in research. Neurogastroenterol. Motil..

[118] Dinning P.G. (2018). A new understanding of the physiology and pathophysiology of colonic motility?. Neurogastroenterol. Motil..

[119] Corsetti M., Costa M., Bassotti G., Bharucha A.E., Borrelli O., Dinning P., Di Lorenzo C., Huizinga J.D., Jimenez M., Rao S. (2019). First translational consensus on terminology and definitions of colonic motility in animals and humans studied by manometric and other techniques. Nat. Rev. Gastroenterol. Hepatol..

[120] Campbell-Thompson M.L., Kaddis J.S., Wasserfall C., Haller M.J., Pugliese A., Schatz D.A., Shuster J.J., Atkinson M.A. (2016). The influence of type 1 diabetes on pancreatic weight. Diabetologia.

[121] Zhao J., Yang J., Gregersen H. (2003). Biomechanical and morphometric intestinal remodelling during experimental diabetes in rats. Diabetologia.

[122] Klinge M.W., Borghammer P., Lund S., Fedorova T., Knudsen K., Haase A.M., Christiansen J.J., Krogh K. (2020). Enteric cholinergic neuropathy in patients with diabetes: Non-invasive assessment with positron emission tomography. Neurogastroenterol. Motil..

[123] Masselli G., Gualdi G. (2012). MR imaging of the small bowel. Radiology.

[124] Frøkjaer J.B., Brock C., Brun J., Simren M., Dimcevski G., Funch-Jensen P., Drewes A.M., Gregersen H. (2012). Esophageal distension parameters as potential biomarkers of impaired gastrointestinal function in diabetes patients. Neurogastroenterol. Motil..

[125] Banerjee S., Pal A., Fox M. (2020). Volume and position change of the stomach during gastric accommodation and emptying: A detailed three-dimensional morphological analysis based on MRI. Neurogastroenterol. Motil..

[126] Menys A., Keszthelyi D., Fitzke H., Fikree A., Atkinson D., Aziz Q., Taylor S.A. (2017). A magnetic resonance imaging study of gastric motor function in patients with dyspepsia associated with Ehlers-Danlos Syndrome-Hypermobility Type: A feasibility study. Neurogastroenterol. Motil..

[127] Lehmann R., Borovicka J., Kunz P., Crelier G., Boesiger P., Fried M., Schwizer W., Spinas G.A. (1996). Evaluation of delayed gastric emptying in diabetic patients with autonomic neuropathy by a new magnetic resonance imaging technique and radio-opaque markers. Diabetes Care.

[128] Carbone S.F., Tanganelli I., Capodivento S., Ricci V., Volterrani L. (2010). Magnetic resonance imaging in the evaluation of the gastric emptying and antral motion: Feasibility and reproducibility of a fast not invasive technique. Eur. J. Radiol..

[129] Cho J., Lee Y.J., Kim Y.H., Shin C.M., Kim J.M., Chang W., Park J.H. (2019). Quantitative MRI evaluation of gastric motility in patients with Parkinson’s disease: Correlation of dyspeptic symptoms with volumetry and motility indices. PLoS ONE.

[130] Unger M.M., Hattemer K., Möller J.C., Schmittinger K., Mankel K., Eggert K., Strauch K., Tebbe J.J., Keil B., Oertel W.H. (2010). Real-time visualization of altered gastric motility by magnetic resonance imaging in patients with Parkinson’s disease. Mov. Disord..

[131] Froehlich J.M., Patak M.A., von Weymarn C., Juli C.F., Zollikofer C.L., Wentz K.U. (2005). Small bowel motility assessment with magnetic resonance imaging. J. Magn Reson. Imaging.

[132] Ajaj W., Goehde S.C., Papanikolaou N., Holtmann G., Ruehm S.G., Debatin J.F., Lauenstein T.C. (2004). Real time high resolution magnetic resonance imaging for the assessment of gastric motility disorders. Gut.

[133] Fedorova T.D., Knudsen K., Hartmann B., Holst J.J., Viborg Mortensen F., Krogh K., Borghammer P. (2020). In vivo positron emission tomography imaging of decreased parasympathetic innervation in the gut of vagotomized patients. Neurogastroenterol. Motil..

[134] Fedorova T.D., Seidelin L.B., Knudsen K., Schacht A.C., Geday J., Pavese N., Brooks D.J., Borghammer P. (2017). Decreased intestinal acetylcholinesterase in early Parkinson disease: An (11)C-donepezil PET study. Neurology.

[135] Gjerløff T., Fedorova T., Knudsen K., Munk O.L., Nahimi A., Jacobsen S., Danielsen E.H., Terkelsen A.J., Hansen J., Pavese N. (2015). Imaging acetylcholinesterase density in peripheral organs in Parkinson’s disease with 11C-donepezil PET. Brain.

[136] Knowles C.H., Lindberg G., Panza E., De Giorgio R. (2013). New perspectives in the diagnosis and management of enteric neuropathies. Nat. Rev. Gastroenterol. Hepatol..

[137] Swaminathan M., Kapur R.P. (2010). Counting myenteric ganglion cells in histologic sections: An empirical approach. Hum. Pathol..

[138] Ippolito C., Segnani C., De Giorgio R., Blandizzi C., Mattii L., Castagna M., Moscato S., Dolfi A., Bernardini N. (2009). Quantitative evaluation of myenteric ganglion cells in normal human left colon: Implications for histopathological analysis. Cell Tissue Res..

[139] Coron E., Auksorius E., Pieretti A., Mahé M.M., Liu L., Steiger C., Bromberg Y., Bouma B., Tearney G., Neunlist M. (2012). Full-field optical coherence microscopy is a novel technique for imaging enteric ganglia in the gastrointestinal tract. Neurogastroenterol. Motil..

[140] Boschetti E., Malagelada C., Accarino A., Malagelada J.R., Cogliandro R.F., Gori A., Bonora E., Giancola F., Bianco F., Tugnoli V. (2019). Enteric neuron density correlates with clinical features of severe gut dysmotility. Am. J. Physiol. Gastrointest. Liver. Physiol..

[141] Selim M.M., Wendelschafer-Crabb G., Redmon J.B., Khoruts A., Hodges J.S., Koch K., Walk D., Kennedy W.R. (2010). Gastric mucosal nerve density: A biomarker for diabetic autonomic neuropathy?. Neurology.

[142] Sprenger F.S., Stefanova N., Gelpi E., Seppi K., Navarro-Otano J., Offner F., Vilas D., Valldeoriola F., Pont-Sunyer C., Aldecoa I. (2015). Enteric nervous system α-synuclein immunoreactivity in idiopathic REM sleep behavior disorder. Neurology.

[143] Lebouvier T., Neunlist M., Bruley des Varannes S., Coron E., Drouard A., N’Guyen J.M., Chaumette T., Tasselli M., Paillusson S., Flamand M. (2010). Colonic biopsies to assess the neuropathology of Parkinson’s disease and its relationship with symptoms. PLoS ONE.

[144] Giancola F., Fracassi F., Gallucci A., Sadeghinezhad J., Polidoro G., Zini E., Asti M., Chiocchetti R. (2016). Quantification of nitrergic neurons in the myenteric plexus of gastric antrum and ileum of healthy and diabetic dogs. Auton. Neurosci..

[145] Blum H.E. (2017). The human microbiome. Adv. Med. Sci..

[146] Fraher M.H., O’Toole P.W., Quigley E.M. (2012). Techniques used to characterize the gut microbiota: A guide for the clinician. Nat. Rev. Gastroenterol. Hepatol..

[147] Marchesi J.R., Adams D.H., Fava F., Hermes G.D., Hirschfield G.M., Hold G., Quraishi M.N., Kinross J., Smidt H., Tuohy K.M. (2016). The gut microbiota and host health: A new clinical frontier. Gut.

[148] Hasegawa S., Goto S., Tsuji H., Okuno T., Asahara T., Nomoto K., Shibata A., Fujisawa Y., Minato T., Okamoto A. (2015). Intestinal Dysbiosis and Lowered Serum Lipopolysaccharide-Binding Protein in Parkinson’s Disease. PLoS ONE.

[149] Keshavarzian A., Green S.J., Engen P.A., Voigt R.M., Naqib A., Forsyth C.B., Mutlu E., Shannon K.M. (2015). Colonic bacterial composition in Parkinson’s disease. Mov. Disord..

[150] Sampson T.R., Debelius J.W., Thron T., Janssen S., Shastri G.G., Ilhan Z.E., Challis C., Schretter C.E., Rocha S., Gradinaru V. (2016). Gut Microbiota Regulate Motor Deficits and Neuroinflammation in a Model of Parkinson’s Disease. Cell.

[151] Unger M.M., Spiegel J., Dillmann K.U., Grundmann D., Philippeit H., Bürmann J., Faßbender K., Schwiertz A., Schäfer K.H. (2016). Short chain fatty acids and gut microbiota differ between patients with Parkinson’s disease and age-matched controls. Parkinsonism Relat. Disord..

[152] Brown C.T., Davis-Richardson A.G., Giongo A., Gano K.A., Crabb D.B., Mukherjee N., Casella G., Drew J.C., Ilonen J., Knip M. (2011). Gut microbiome metagenomics analysis suggests a functional model for the development of autoimmunity for type 1 diabetes. PLoS ONE.

[153] Giongo A., Gano K.A., Crabb D.B., Mukherjee N., Novelo L.L., Casella G., Drew J.C., Ilonen J., Knip M., Hyöty H. (2011). Toward defining the autoimmune microbiome for type 1 diabetes. ISME J..

[154] Siljander H., Honkanen J., Knip M. (2019). Microbiome and type 1 diabetes. EBioMedicine.

[155] Miranda M.C.G., Oliveira R.P., Torres L., Aguiar S.L.F., Pinheiro-Rosa N., Lemos L., Guimarães M.A., Reis D., Silveira T., Ferreira Ê. (2019). Frontline Science: Abnormalities in the gut mucosa of non-obese diabetic mice precede the onset of type 1 diabetes. J. Leukoc. Biol..

[156] Vaarala O., Atkinson M.A., Neu J. (2008). The “perfect storm” for type 1 diabetes: The complex interplay between intestinal microbiota, gut permeability, and mucosal immunity. Diabetes.

[157] Cani P.D. (2019). Microbiota and metabolites in metabolic diseases. Nat. Rev. Endocrinol..

[158] Softeland E., Brock C., Frokjaer J.B., Brogger J., Madacsy L., Gilja O.H., Arendt-Nielsen L., Simren M., Drewes A.M., Dimcevski G. (2014). Association between visceral, cardiac and sensorimotor polyneuropathies in diabetes mellitus. J. Diabetes Complicat..

[159] Pop-Busui R., Boulton A.J., Feldman E.L., Bril V., Freeman R., Malik R.A., Sosenko J.M., Ziegler D. (2017). Diabetic Neuropathy: A Position Statement by the American Diabetes Association. Diabetes Care.

[160] Low P.A., Denq J.C., Opfer-Gehrking T.L., Dyck P.J., O’Brien P.C., Slezak J.M. (1997). Effect of age and gender on sudomotor and cardiovagal function and blood pressure response to tilt in normal subjects. Muscle Nerve.

[161] Sandroni P., Benarroch E.E., Low P.A. (1991). Pharmacological dissection of components of the Valsalva maneuver in adrenergic failure. J. Appl. Physiol..

[162] Benarroch E.E., Opfer-Gehrking T.L., Low P.A. (1991). Use of the photoplethysmographic technique to analyze the Valsalva maneuver in normal man. Muscle Nerve.

[163] Freeman R., Chapleau M.W. (2013). Testing the autonomic nervous system. Handb. Clin. Neurol..

[164] Spallone V. (2018). Blood Pressure Variability and Autonomic Dysfunction. Curr. Diabetes Rep..

[165] Freeman R., Wieling W., Axelrod F.B., Benditt D.G., Benarroch E., Biaggioni I., Cheshire W.P., Chelimsky T., Cortelli P., Gibbons C.H. (2011). Consensus statement on the definition of orthostatic hypotension, neurally mediated syncope and the postural tachycardia syndrome. Clin. Auton. Res..

[166] Cheshire W.P. (2020). Autonomic History, Examination, and Laboratory Evaluation. Continuum.

[167] Freeman R. (2020). Autonomic Peripheral Neuropathy. Continuum.

[168] Spallone V., Ziegler D., Freeman R., Bernardi L., Frontoni S., Pop-Busui R., Stevens M., Kempler P., Hilsted J., Tesfaye S. (2011). Cardiovascular autonomic neuropathy in diabetes: Clinical impact, assessment, diagnosis, and management. Diabetes Metab Res. Rev..

[169] Sletten D.M., Weigand S.D., Low P.A. (2010). Relationship of Q-sweat to quantitative sudomotor axon reflex test (QSART) volumes. Muscle Nerve.

[170] Schwartz T.W. (1983). Pancreatic polypeptide: A unique model for vagal control of endocrine systems. J. Auton. Nerv. Syst..

[171] Knudsen K., Hartmann B., Fedorova T.D., Østergaard K., Krogh K., Møller N., Holst J.J., Borghammer P. (2017). Pancreatic Polypeptide in Parkinson’s Disease: A Potential Marker of Parasympathetic Denervation. J. Park. Dis..

[172] Desai A., Low P.A., Camilleri M., Singer W., Burton D., Chakraborty S., Bharucha A.E. (2020). Utility of the plasma pancreatic polypeptide response to modified sham feeding in diabetic gastroenteropathy and non-ulcer dyspepsia. Neurogastroenterol. Motil..

[173] Parkman H.P., Yates K.P., Hasler W.L., Nguyan L., Pasricha P.J., Snape W.J., Farrugia G., Calles J., Koch K.L., Abell T.L. (2011). Dietary intake and nutritional deficiencies in patients with diabetic or idiopathic gastroparesis. Gastroenterology.

[174] Astarloa R., Mena M.A., Sánchez V., de la Vega L., de Yébenes J.G. (1992). Clinical and pharmacokinetic effects of a diet rich in insoluble fiber on Parkinson disease. Clin. Neuropharmacol..

[175] Jalleh R., Marathe C.S., Rayner C.K., Jones K.L., Horowitz M. (2019). Diabetic Gastroparesis and Glycaemic Control. Curr. Diabetes Rep..

[176] Acosta A., Camilleri M. (2015). Prokinetics in gastroparesis. Gastroenterol. Clin. N. Am..

[177] Giudicessi J.R., Ackerman M.J., Camilleri M. (2018). Cardiovascular safety of prokinetic agents: A focus on drug-induced arrhythmias. Neurogastroenterol. Motil..

[178] Kumar M., Chapman A., Javed S., Alam U., Malik R.A., Azmi S. (2018). The Investigation and Treatment of Diabetic Gastroparesis. Clin. Ther..

[179] Iodice V., Kimpinski K., Vernino S., Sandroni P., Low P.A. (2009). Immunotherapy for autoimmune autonomic ganglionopathy. Auton. Neurosci..

[180] McCallum R.W., Lin Z., Forster J., Roeser K., Hou Q., Sarosiek I. (2011). Gastric electrical stimulation improves outcomes of patients with gastroparesis for up to 10 years. Clin. Gastroenterol. Hepatol..

[181] Klinge M.W., Rask P., Mortensen L.S., Lassen K., Ejskjaer N., Ehlers L.H., Krogh K. (2017). Early Assessment of Cost-effectiveness of Gastric Electrical Stimulation for Diabetic Nausea and Vomiting. J. Neurogastroenterol. Motil..

[182] Okdahl T., Bertoli D., Brock B., Krogh K., Krag Knop F., Brock C., Drewes A.M. (2021). Study protocol for a multicentre, randomised, parallel group, sham-controlled clinical trial investigating the effect of transcutaneous vagal nerve stimulation on gastrointestinal symptoms in people with diabetes complicated with diabetic autonomic neuropathy: The DAN-VNS Study. BMJ Open.

[183] Lauritano E.C., Gabrielli M., Scarpellini E., Lupascu A., Novi M., Sottili S., Vitale G., Cesario V., Serricchio M., Cammarota G. (2008). Small intestinal bacterial overgrowth recurrence after antibiotic therapy. Am. J. Gastroenterol..

[184] Selby A., Reichenbach Z.W., Piech G., Friedenberg F.K. (2019). Pathophysiology, Differential Diagnosis, and Treatment of Diabetic Diarrhea. Dig. Dis. Sci..

